# Leukocyte integrin Mac-1 regulates thrombosis via interaction with platelet GPIbα

**DOI:** 10.1038/ncomms15559

**Published:** 2017-05-30

**Authors:** Yunmei Wang, Huiyun Gao, Can Shi, Paul W. Erhardt, Alexander Pavlovsky, Dmitry A. Soloviev, Kamila Bledzka, Valentin Ustinov, Liang Zhu, Jun Qin, Adam D. Munday, Jose Lopez, Edward Plow, Daniel I. Simon

**Affiliations:** 1 Case Cardiovascular Research Institute, Case Western Reserve University School of Medicine and Harrington Heart & Vascular Institute, University Hospitals Cleveland Medical Center, Cleveland, Ohio 44106, USA; 2 Department of Medicinal and Biological Chemistry, University of Toledo College of Pharmacy and Pharmaceutical Sciences, Toledo, Ohio 43614, USA; 3 Department of Molecular Cardiology, Cleveland Clinic, Cleveland, Ohio 44106, USA; 4 Bloodworks Northwest Research Institute, Seattle, Washington 98102, USA

## Abstract

Inflammation and thrombosis occur together in many diseases. The leukocyte integrin Mac-1 (also known as integrin α_M_β_2_, or CD11b/CD18) is crucial for leukocyte recruitment to the endothelium, and Mac-1 engagement of platelet GPIbα is required for injury responses in diverse disease models. However, the role of Mac-1 in thrombosis is undefined. Here we report that mice with Mac-1 deficiency (*Mac-1*^*−/−*^) or mutation of the Mac-1-binding site for GPIbα have delayed thrombosis after carotid artery and cremaster microvascular injury without affecting parameters of haemostasis. Adoptive wild-type leukocyte transfer rescues the thrombosis defect in *Mac-1*^*−/−*^ mice, and Mac-1-dependent regulation of the transcription factor Foxp1 contributes to thrombosis as evidenced by delayed thrombosis in mice with monocyte-/macrophage-specific overexpression of Foxp1. Antibody and small-molecule targeting of Mac-1:GPIbα inhibits thrombosis. Our data identify a new pathway of thrombosis involving leukocyte Mac-1 and platelet GPIbα, and suggest that targeting this interaction has anti-thrombotic therapeutic potential with reduced bleeding risk.

## Abstract (web summary)

The binding of the leukocyte integrin Mac1 to the platelet receptor GPIbα is important for the physiological response to tissue injury. Here the authors show that this interaction also regulates thrombosis, without influencing bleeding time, which may provide clues for the development of new anti-thrombotic drugs.

Thrombotic cardiovascular diseases, including myocardial infarction and stroke, are the leading cause of death in developed countries^1^. Current anti-thrombotic drugs, including antiplatelet agents and anticoagulants, are associated with significant bleeding risk and increased mortality^2^,^3^,^4^. There is emerging experimental evidence distinguishing the molecular and cellular mechanisms of haemostasis and thrombosis^5^,^6^, thereby providing potential therapeutic targets with reduced bleeding risk. One such area of research focus is ligand–receptor interactions, including CD40L and its binding to platelet GPIIb/IIIa (ref. 7), Gas6 and its tyrosine kinase receptors (mer, tyro3 and axl)^8^, ephrins and their eph kinase receptors^9^, and myeloid-related protein-8/14 (MRP-8/14 or S100A8/A9) and its platelet CD36 receptor^10^, that act within the platelet–platelet contact zone or ‘synapse’ after the initial aggregation event and ultimately promote thrombus growth and stability^11^.

A second area of research focus that distinguishes molecular and cellular mechanisms of haemostasis and thrombosis involves heterotypic cell–cell interactions between leukocytes and platelets. Platelet–leukocyte interactions induce bidirectional signals that amplify pro-inflammatory and pro-thrombotic cellular responses^12^. A more complete understanding of the molecular basis of leukocyte–platelet complex formation may provide key insight into candidate anti-thrombotic targets.

Adhesive interactions between vascular cells play important roles in orchestrating the inflammatory response. Recruitment of circulating leukocytes to vascular endothelium requires multistep adhesive and signalling events, including selectin-mediated attachment and rolling, leukocyte activation, and integrin-mediated firm adhesion and diapedesis that result in the infiltration of inflammatory cells into the blood vessel wall^13^. Firm attachment is mediated by members of the β_2_-integrin family, LFA-1 (α_L_β_2_, CD11a/CD18), Mac-1 (α_M_β_2_, CD11b/CD18) and p150,95 (α_x_β_2_, CD11c/CD18), and CD11d/CD18 (α_D_β_2_), which bind to endothelial counter ligands (for example, intercellular adhesion molecule-1; ICAM-1), endothelial-associated extracellular matrix proteins (for example, fibrinogen) or glycosaminoglycans^14^,^15^.

Leukocyte recruitment and infiltration also occur at sites of vascular injury where the lining endothelial cells have been denuded, and platelets and fibrin have been deposited. A similar sequential adhesion model of leukocyte attachment to and transmigration across surface-adherent platelets has been proposed^16^. The initial tethering and rolling of leukocytes on platelet P-selectin^17^ are followed by their firm adhesion and transplatelet migration, processes that are dependent on α_M_β_2_ (ref. 16).

Integrins are heterodimeric proteins composed of one α- and one β-subunit. A subset of integrin α-subunits, including α_M_, contains an inserted domain (I-domain) of ∼200 amino acids that is implicated in ligand binding^18^ and is strikingly similar to the A domains of von Willebrand factor (vWF)^19^, one of which, A1, mediates the interaction of vWF with its platelet receptor, the glycoprotein (GP) Ib–IX–V complex. Because of the similarity of the vWF A1 domain and the α_M_I-domain, we hypothesized that GPIbα might also be able to bind α_M_β_2_ and reported that GPIbα is indeed a constitutively expressed counterreceptor for α_M_β_2_ (ref. 20).

The α_M_I-domain contributes broadly to the recognition of ligands by α_M_β_2_ (ref. 18) and specifically to the binding of GPIbα (ref. 20). This region has also been implicated in the binding of many ligands, including ICAM-1 (ref. 21), C3bi (ref. 22) and fibrinogen^21^. We localized the binding site for GPIbα within the α_M_I-domain segment α_M_(P^201^–K^217^) using a strategy based on the differences in the binding of GPIbα to the α_M_I- and α_L_I-domains that involved several independent approaches, including screening of mutant cells, synthetic peptides, site-directed mutagenesis and gain-in-function analyses^23^. Antibody targeting of α_M_(P^201^–K^217^) blocked α_M_β_2_-dependent adhesion to GPIbα, but not several other ligands and inhibited leukocyte accumulation, cellular proliferation and neointimal thickening after arterial injury^24^, and broadly regulated the biological response to tissue injury in models of vasculitis^25^, glomerulonephritis^26^ and experimental autoimmune encephalomyelitis^27^.

Since leukocyte–platelet interactions bidirectionally induce signals that amplify pro-inflammatory and pro-thrombotic cellular responses^12^, we hypothesized that leukocyte Mac-1 engagement of platelet GPIbα is critical for thrombus formation. In this study utilizing genetic, antibody, and small-molecule approaches, we provide evidence that Mac-1:GPIbα directly modulates thrombosis without influence on tail bleeding time or other haemostatic parameters.

## Results

### Carotid artery thrombosis is delayed in *Mac-1* ^*−/−*^ mice

**Figure 1:**
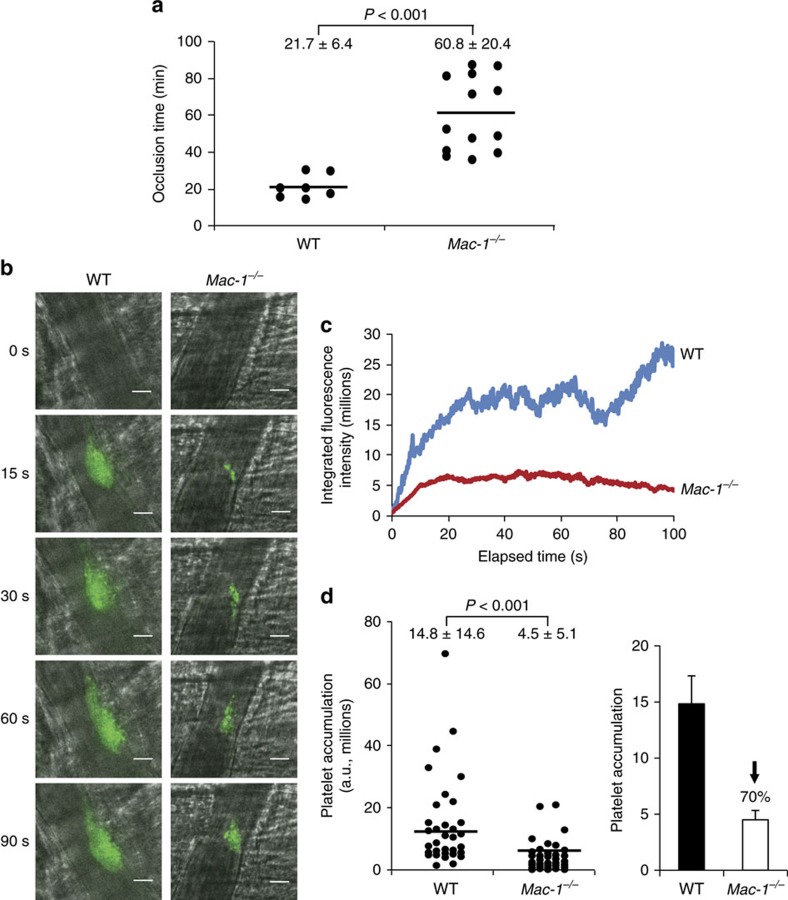
Deficiency of Mac-1 prolongs thrombotic occlusion time. (**a**) Occlusion time (min) following photochemical injury of the carotid artery in 7- to 8-week-old male WT (*n*=7) and *Mac-1*^*−/−*^ (*n*=13) mice (mean±s.d.). Thrombus formation after laser-induced injury to the arteriolar wall of the cremaster microvasculature of *Mac-1*^*−/−*^ mice was compared with that of WT mice using intravital microscopy (**b**–**d**). Platelets were labelled *in vivo* using a fluorescein isothiocyanate-conjugated rat anti-mouse CD41 antibody. (**b**) Representative intravital images at indicated times following laser pulse (*n*=33–35 per group). Scale bar, 20 μm. (**c**) Continuous, real-time thrombosis profiles of one representative experiment (*n*=33–35 arterioles per group). (**d**) Mean fluorescence intensity of platelets in individual arterioles over time. Each data point is from a single arteriole. Data (mean±s.d.) taken from four WT and four *Mac-1*^*−/−*^ 11- to 12-week-old male mice. *P* values are obtained by two-tailed unpaired *t-*test.

To elucidate the effect of Mac-1 on the development of arterial thrombosis in real-time, carotid arteries of wild-type (WT) and *Mac-1*^*−/−*^ mice were subjected to the Rose Bengal model of thrombosis, an endothelial cell photochemical injury model due to local free radical release^28^,^29^. Carotid artery blood flow was then monitored continuously with a vascular flow probe. Mean time to occlusive thrombus formation in WT mice was 21.7±6.4 min, and was prolonged significantly in *Mac-1*^*−/−*^ mice to 60.8±20.4 min (*n*=7–13 per group; Fig. 1a).

### Impaired thrombus formation in *Mac-1* ^*−/−*^ microvasculature

The cremaster thrombosis model was implemented to examine the influence of Mac-1 in a small vessel arteriole as contrasted to the large vessel carotid artery used in the Rose Bengal model. Thrombus formation after laser-induced injury to the arteriolar wall in the cremaster microcirculation of *Mac-1*^*−/−*^ mice was compared with that of WT mice, using intravital microscopy^30^. In WT mice, platelet accumulation in arterioles was evident within 15 s of laser injury and increased progressively over the 90 s observation period (Fig. 1b). In contrast, platelet accumulation was markedly attenuated in *Mac-1*^*−/−*^ mice. Analysis of the growth curves of continuous, real-time thrombus profiles assessed by integrated fluorescence intensity of labelled platelets over time showed marked attenuation of platelet thrombus growth in *Mac-1*^*−/−*^ mice (WT: 14.8±14.6 × 10^6^ arbitrary fluorescent units (a.u.) versus *Mac-1*^*−/−*^: 4.5±5.1 × 10^6^ a.u., *n*=33–35 arterioles per group; Fig. 1c,d). Mean % inhibition over time was 70.0%. Initial platelet adhesion and small platelet aggregates were observed in *Mac-1*^*−/−*^mice, but developing thrombi were unstable and embolized frequently.

### Similar platelet count and coagulation assays in WT and *Mac-1* ^*−/−*^ mice

**Figure 2:**
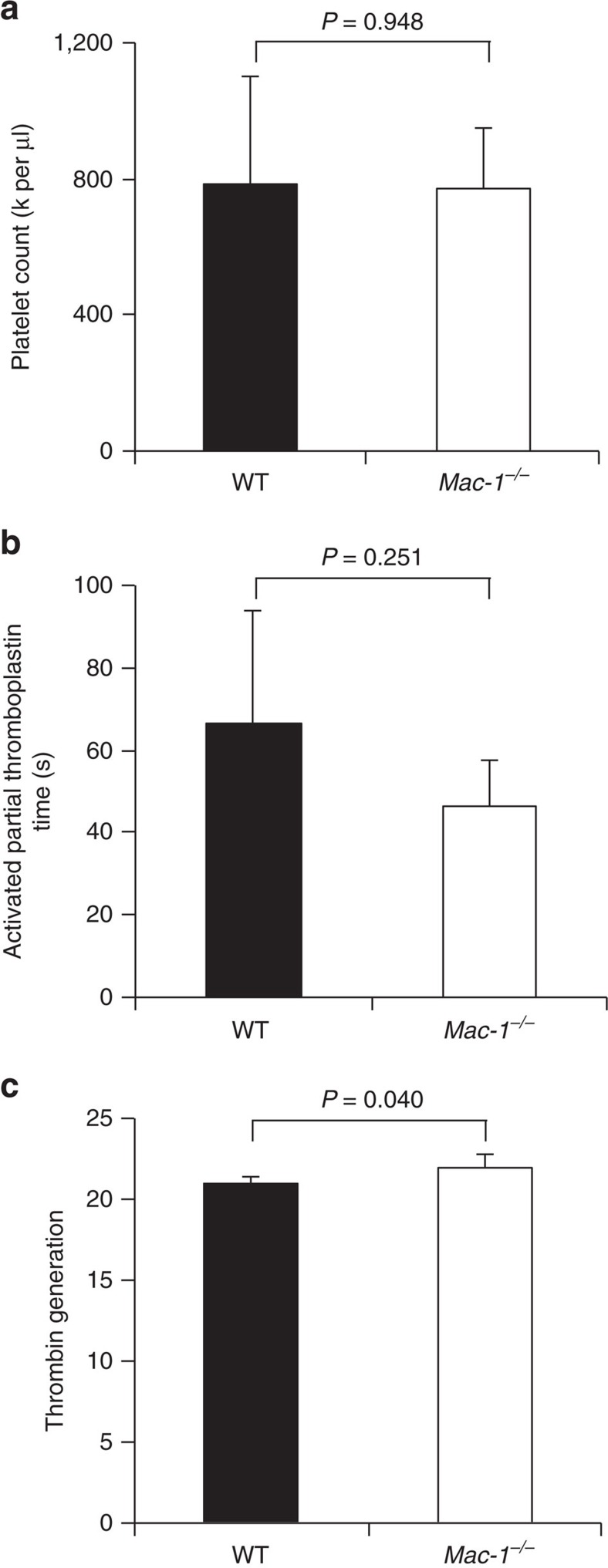
Deficiency of Mac-1 has no influence on platelet count or coagulation activity. (**a**) Platelet count (10^3^ μl^−1^) of citrate-anticoagulated mouse blood obtained from 8-week-old male WT (*n*=5) or *Mac-1*^*−/−*^ mice (*n*=6). (**b**) aPTT (s) in WT and *Mac-1*^*−/−*^ mice (*n*=4 per group). (**c**) TF-induced thrombin generation was assayed using plasma of WT and *Mac-1*^*−/−*^ mice (*n*=4 per group; mean±s.d.). *P* values are obtained by unpaired two-tailed *t-*test.

Having observed delayed thrombosis in *Mac-1*^*−/−*^ mice, we set out to determine the mechanism by first performing screening platelet and coagulation assays in WT and *Mac-1*^*−/−*^ mice. We performed complete blood count on whole blood from WT and *Mac-1*^*−/−*^ mice (Supplementary Table 1). White blood cell count (Supplementary Table 1) and platelet count were similar in WT (786,400±317,800 platelets per μl, *n*=5) and in *Mac-1*^*−/−*^ (775,700±176,000 platelets per μl, *n*=6) mice (Fig. 2a). Coagulation activity of plasma was assessed using the activated partial thromboplastin time (aPTT) and a thrombin generation assay. The aPTT was not prolonged in *Mac-1*^*−/−*^ mice (WT: 66±28 s versus *Mac-1*^*−/−*^: 46±11 s; Fig. 2b). Tissue factor (TF)-induced total thrombin generation was not reduced in *Mac-1*^*−/−*^ plasma (WT: 21,055±407 versus *Mac-1*^*−/−*^: 22,398±830 a.u., *n*=4 per group; Fig. 2c). Taken together, these data indicate that neither platelet count nor coagulation parameters likely account for delayed thrombosis in *Mac-1*^*−/−*^ mice.

### Unimpaired platelet activation and signalling in *Mac-1* ^*−/−*^ platelets

Although leukocyte-restricted expression of Mac-1 makes it unlikely that defective thrombus formation in *Mac-1*^*−/−*^ mice is due to an intrinsic platelet activation defect, we nonetheless assessed platelet activation in platelets isolated from WT and *Mac-1*^*−/−*^ mice by monitoring the expression of P-selectin and activated α_IIb_β_3_ (GPIIb/IIIa) in response to agonist stimulation. Washed platelets from WT and *Mac-1*^*−/−*^ mice were stimulated with collagen, thrombin or arachidonic acid. Platelet activation as measured by both P-selectin expression (Wug.E9-positive staining) and activated α_IIb_β_3_ (JON/A-positive staining) is similar in *Mac-1*^*−/−*^ platelets compared to WT platelets in response to the treatment of thrombin (Supplementary Fig. 1a,b), collagen (Supplementary Fig. 1c,d) or arachidonic acid (Supplementary Fig. 1e,f). In addition, we demonstrated there was no difference in platelet adhesion (Supplementary Fig. 1g) and spreading on collagen (Supplementary Fig. 1h) between WT and *Mac-1*^*−/−*^ mice.

### Transfer of WT leukocytes restores thrombosis defect in *Mac-1* ^*−/−*^ mice

**Figure 3:**
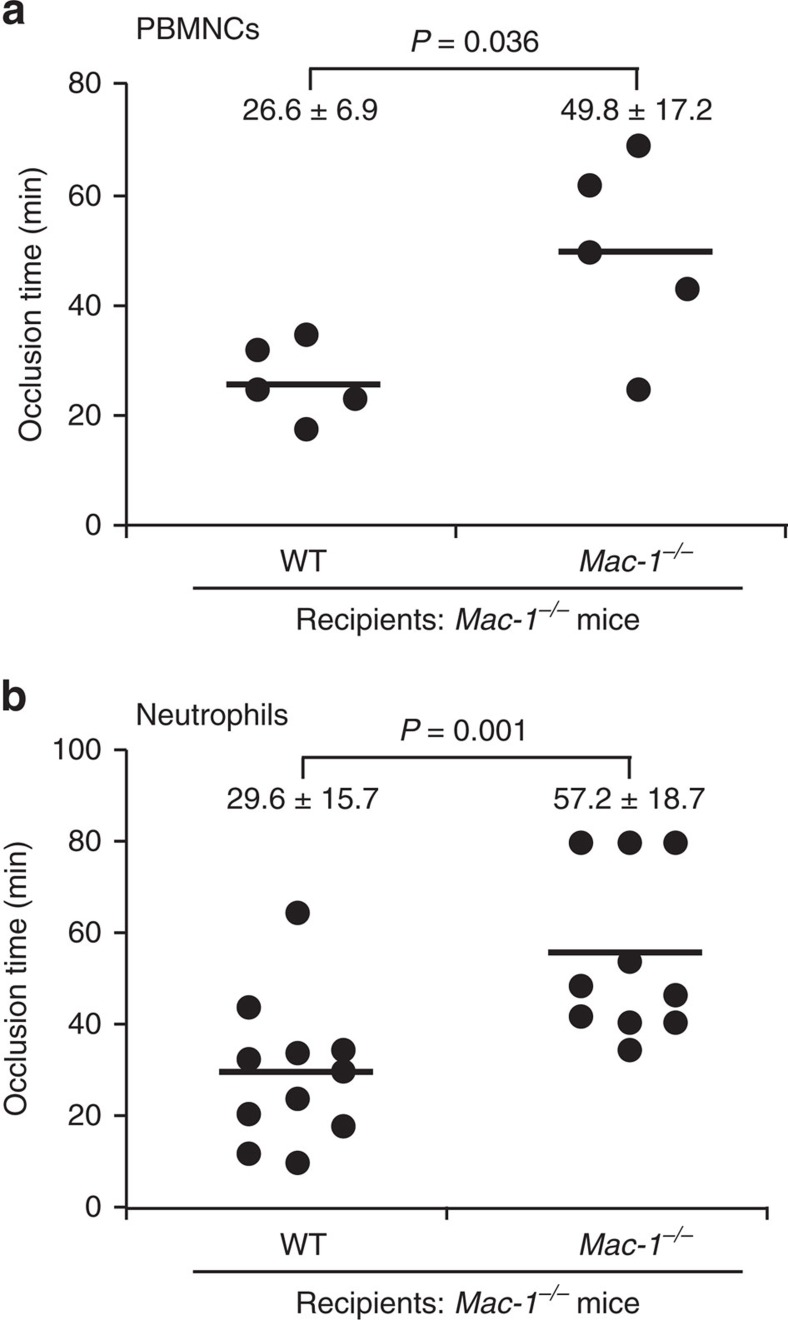
Adoptive transfer of WT leukocytes rescues the thrombus defect in *Mac-1*^*−/−*^ mice. Thrombotic occlusion times in *Mac-1*^*−/−*^ recipient mice undergoing adoptive transfer of 1 × 10^6^ WT or *Mac-1*^*−/−*^ PBMCs (**a**) or neutrophils (**b**) via tail vein injection before carotid artery photochemical injury (mean±s.d.). Each data point is from one mouse received WT or *Mac-1*^*−/−*^ PBMCs or neutrophils as indicated. *P* values are obtained by unpaired two-tailed *t-*test.

Although we have provided evidence that deficiency of Mac-1 is associated with prolonged time to carotid artery occlusion after photochemical injury, we have utilized mice with global rather than tissue- or cell-specific deficiency of Mac-1, thereby limiting our ability to conclude definitively that leukocyte Mac-1 is critical for thrombus formation. To address this issue, we performed adoptive transfer experiments of WT and *Mac-1*^*−/−*^ donor peripheral blood mononuclear cells (PBMCs) or neutrophils into *Mac-1*^*−/−*^ recipient mice before photochemical injury. *Mac-1*^*−/−*^ recipient mice that received *Mac-1*^*−/−*^ donor PBMCs formed occlusive thrombi in 49.8±17.2 min (Fig. 3a). Strikingly, the time to occlusive thrombus formation was shortened significantly in *Mac-1*^*−/−*^ recipient mice receiving WT donor PBMCs to 26.6±6.9 min (Fig. 3a), nearly restoring occlusion time to that observed in WT mice (21.7±6.4 min; Fig. 1a).

We also performed adoptive transfer of WT or *Mac-1*^*−/−*^ neutrophils isolated by density gradient centrifugation into *Mac-1*^*−/−*^ recipient mice. *Mac-1*^*−/−*^ mice that received *Mac-1*^*−/−*^ donor neutrophils formed occlusive thrombi in 57.2±18.7 min. The time to occlusive thrombus formation was significantly shortened in *Mac-1*^*−/−*^ recipient mice that received WT donor neutrophils to 29.6±15.7 min (Fig. 3b). Taken together, these adoptive transfer experiments indicate that Mac-1 on either PBMCs or neutrophils contributes to arterial thrombus formation.

### Mac-1 signalling regulates TF via transcription factor Foxp1

**Figure 4:**
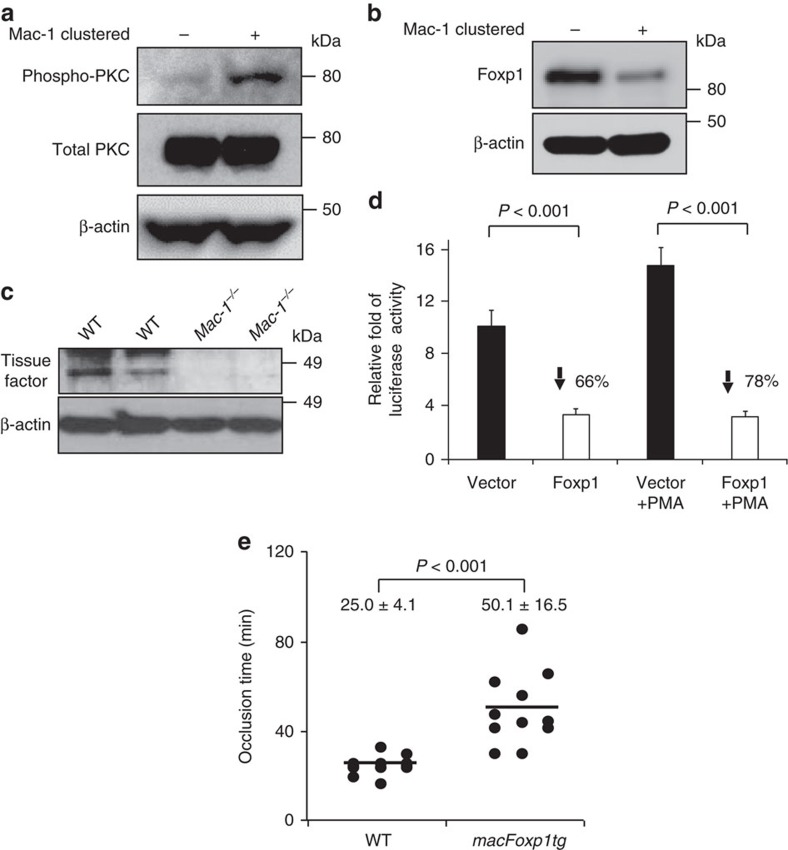
‘Outside-in’ Mac-1 signalling. (**a**) Phosphorylation of PKC-delta in Mac-1-clustered (+) and non-clustered (−) THP-1 monocytic cells was examined by immunoblotting. (**b**) Expression of Foxp1 in Mac-1-clustered (+) and non-clustered (−) THP-1 monocytic cells was examined by immunoblotting. (**c**) TF expression of lipopolysaccharide-stimulated WT and *Mac-1*^*−/−*^ leukocytes was examined by western analysis using anti-TF and anti-β-actin antibodies (loading control). Full, uncropped blots for a, b and c can be found in Supplementary Fig.8. (**d**) TF expression in unstimulated and phorbol 12-myristate 13-acetate (PMA)-stimulated murine NIH/3T3 cells transfected with either Foxp1 or vector control pcDNA was quantified using a TF promoter activity assay. Data shown (mean±s.d.) is from one of two independent experiments, and each experiment was performed in triplicate with three transfection wells. (**e**) Occlusion times following photochemical injury of the carotid artery in WT (*n*=12) and *macFoxp1tg* (*n*=11) mice (mean±s.d.). *P* values are obtained by unpaired two-tailed *t-*test.

Having demonstrated the importance of leukocytes in thrombosis, we next sought to elucidate the underlying mechanism. Platelet–leukocyte interactions bidirectionally induce signals that amplify pro-inflammatory and pro-thrombotic cellular responses^12^. Previous work from our laboratories has demonstrated that leukocyte engagement of platelet GPIbα via Mac-1 induces platelet ‘outside-in’ signalling and platelet activation^31^. We next asked whether leukocyte engagement of platelet GPIbα via Mac-1 is capable of inducing ‘outside-in’ Mac-1 signalling by evaluating phosphorylation of protein kinase C (PKC) and expression of Foxp1. We and others have reported that Mac-1 clustering by fibrinogen triggers phosphorylation and activation of PKC delta that, in turn, regulates expression of the transcription factor Foxp1 (refs 32, 33). Indeed, clustering of Mac-1 by GPIbα induced phosphorylation of PKC delta (Fig. 4a), and downregulated Foxp1 expression (Fig. 4b).

Foxp1 functions as a transcriptional repressor, and we have shown that downregulation of Foxp1 is required for monocyte differentiation and macrophage function *in vitro* and *in vivo*^32^,^34^. We hypothesized that Mac-1 may regulate thrombosis via Foxp1 and TF. We turned our attention to TF because TF initiates arterial thrombus formation in response to laser-induced injury of endothelial cells^35^,^36^. We first analysed the levels of TF in PBMCs isolated from WT and *Mac-1*^*−/−*^ mice. The expression of TF is reduced in *Mac-1*^*−/−*^ compared to WT leukocytes (Fig. 4c). To investigate whether TF expression is influenced by Mac-1–Foxp1 signalling, mouse NIH/3T3 cells were co-transfected with the TF promoter reporter gene plasmid, pRSV-β-gal, and expression plasmids for Foxp1 or pcDNA3.1 vector control. Overexpression of Foxp1 significantly inhibited TF promoter activity in unstimulated (% inhibition=66) and phorbol 12-myristate 13-acetate-stimulated (% inhibition=78) cells (Fig. 4d).

To determine whether Mac-1–Foxp1 signalling plays a critical role in regulating thrombosis *in vivo*, we subjected mice with monocyte-/macrophage-specific overexpression of Foxp1 (*macFoxp1tg*)^34^ and WT control mice to carotid artery photochemical injury. Enforced overexpression of Foxp1 significantly prolonged the time to occlusive thrombus formation from 25.0±4.1 min in WT control to 50.1±16.5 min in *macFoxp1tg* mice (*n*=11–12 per group; Fig. 4e). Interestingly, TF expression was reduced in *Mac-1*^*−/−*^ compared to WT mice (Supplementary Fig. 2). Hence, the prolonged occlusion time in the *Mac-1*^*−/−*^ mice can be attributed, likely in part, to increased Foxp1 expression and, consequently, reduced TF expression.

### Haemostasis is unimpaired in *Mac-1* ^*−/−*^ mice

**Figure 5:**
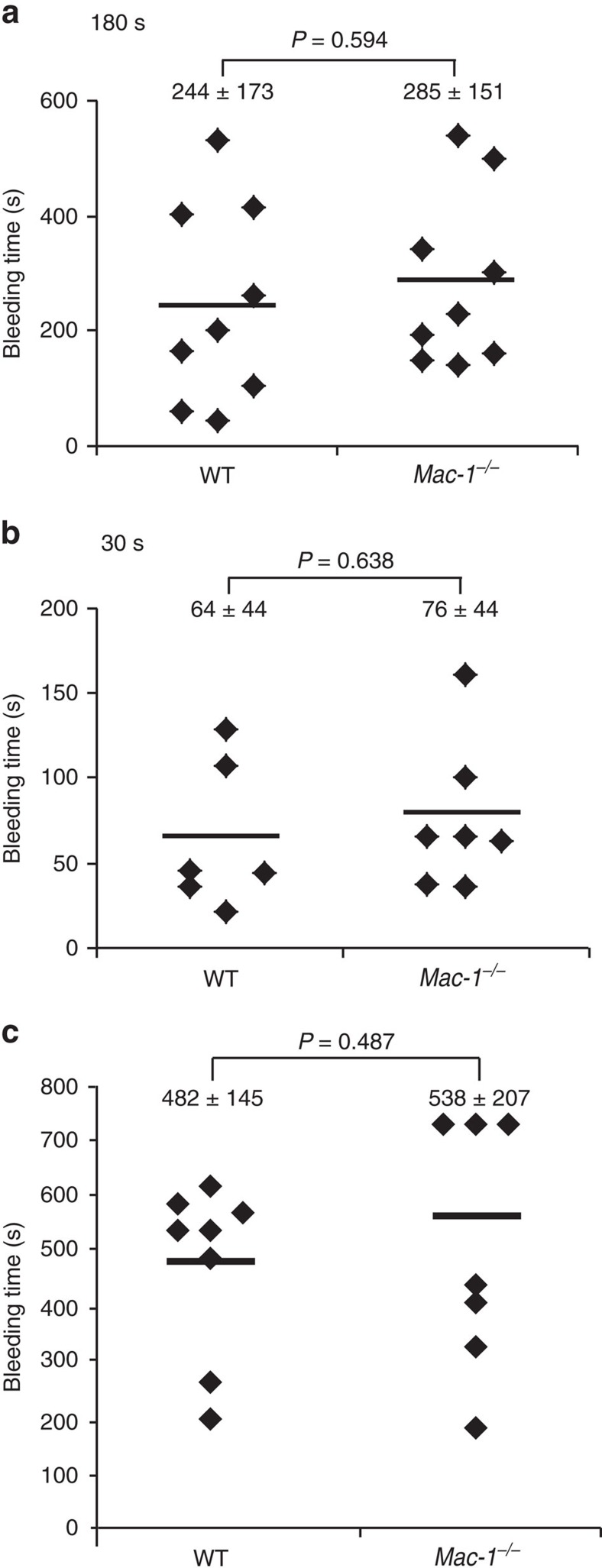
Deficiency of Mac-1 has no effect on tail bleeding time. To assess the role of Mac-1 in haemostasis, tail bleeding times were determined in 2- to 3-month-old WT and *Mac-1*^*−/−*^ male and female mice using two methods. Method 1: the transected tail tip was placed into a beaker containing saline and the time for complete cessation of bleeding either for 3 min (**a**) or 30 s (**b**) was recorded. Method 2: the transected tail tip was blotted with filter paper and the time to complete cessation of bleeding was recorded (**c**). Mean±s.d. (*n*=6–9 per group, each dot represents one data point from one single mouse). *P* values are obtained by unpaired two-tailed *t-*test.

To assess the role of Mac-1 in haemostasis, we examined tail vein bleeding times. When placing the transected tail tip into a beaker containing saline at 37 °C and then determining the time to complete cessation of bleeding, there was no difference in tail bleeding times between WT and *Mac-1*^*−/−*^mice using either complete cessation of bleeding for 3 min or 30 s as the criteria for bleeding time determination. Mean bleeding time for WT mice was 244±173 s compared to 285±151 s for *Mac-1*^*−/−*^ mice (*n*=9 per group) when complete absence of bleeding for 3 min was the end point (Fig. 5a). With a shorter bleeding cessation period of 30 s, the bleeding time in *Mac-1*^*−/−*^ mice was also similar to that in WT mice (76±44 versus 64±44 s; Fig. 5b). Similarly, when blotting the transected tail tip with filter paper and then determining the time to complete cessation of bleeding, there was no difference in tail bleeding times between WT and *Mac-1*^*−/−*^ mice. Mean bleeding time for WT mice was 482±145 s compared to 538±207 s for *Mac-1*^*−/−*^ mice (*n*=7–8 per group; Fig. 5c).

### Mac-1 and GPIbα interaction is critical for regulation of thrombosis

Mac-1, the most abundant β_2_-integrin on neutrophils and monocytes, is a highly promiscuous receptor, capable of binding a broad repertoire of ligands (reviewed in ref. 15) and facilitating key leukocyte functions, including migration, coagulation, proteolysis, phagocytosis, oxidative burst and signalling^14^,^37^,^38^,^39^,^40^. Elegant structural studies by several groups have begun to elucidate the molecular basis for such broad ligand recognition by Mac-1. A subset of integrin α-subunits, including α_M_ of Mac-1, contains an inserted domain (I-domain) of ∼200 amino acids that is implicated in ligand binding. The α_M_I-domain contributes broadly to the recognition of ligands by α_M_β_2_ (refs 18, 41), and the binding sites for C3bi, neutrophil inhibitory factor, fibrinogen and GPIbα have been mapped extensively^23^,^42^,^43^,^44^,^45^,^46^. These studies suggest that overlapping, but not identical, sites are involved in the recognition of C3bi, fibrinogen, neutrophil inhibitory factor and GPIbα (refs 23, 47). We reported previously that the P^201^–K^217^ sequence, which spans an exposed loop and amphipathic α4 helix in the three-dimensional structure of the human α_M_I-domain, was a binding site for GPIbα (ref. 23). Site-directed mutagenesis of the P^201^–K^217^ sequence within the human α_M_I-domain allowed us to further narrow the binding region to H^210^–K^217^ and subsequently identified two single mutants showing reduced binding to GPIbα (T^213^ and R^216^). Indeed, grafting these two critical amino acids onto α_L_ (G^213^T and N^216^R) converted α_L_β_2_ into a GPIbα-binding integrin. Thus, the P^201^–K^217^ sequence within the α_M_I-domain is necessary and sufficient for GPIbα binding. Importantly, this site appears to be highly selective for the binding of GPIbα since antibody targeting the P^201^–K^217^ sequence inhibited the binding of GPIbα, but not other Mac-1 ligands, including fibrinogen, ICAM-1 and junctional adhesion molecule-3 (JAM-3).

**Figure 6:**
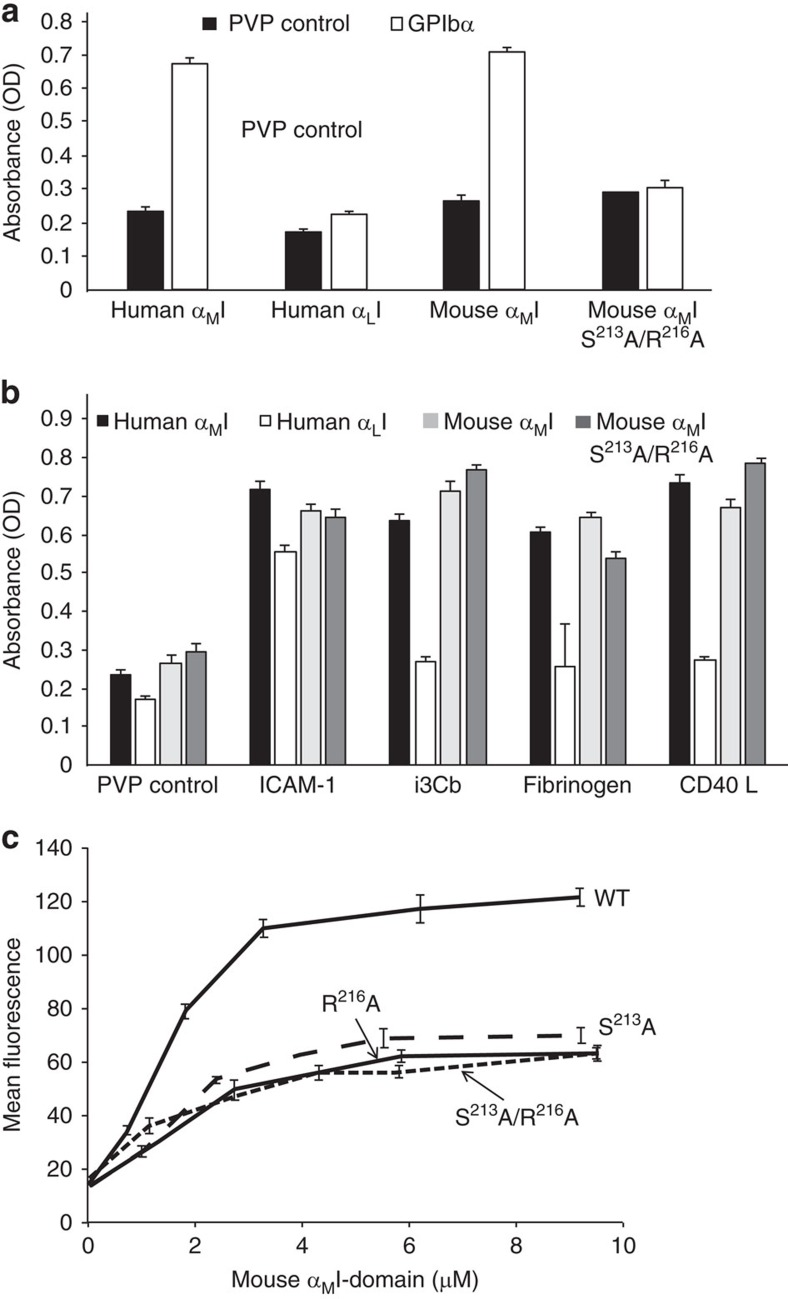
α_M_I-domain binding to GPIbα and generation of muMac-1 (S^213^A/R^216^A) mice. (**a**) Binding of human α_M_ and α_L_I-domains to soluble form of GPIbα was assessed in a solid phase assay. GPIbα was immobilized onto microtitre plates and wells were post-coated with PVP. After washing, the indicated I-domains were allowed to bind for 60 min at 37 °C, plates were washed and binding was detected with a HRP-labelled anti-GST (see Methods). (**b**) Binding of the various α_M_I-domain to the indicated immobilized ligands was detected as in **a**. α_M_ and α_L_I-domains bind to different sites in ICAM-1; the binding of α_L_I-domain to ICAM-1 establishes its functionality. (**c**) Mouse α_M_I-domain fragments, WT, S^213^A, R^216^A and S^213^A/R^216^A, were expressed as GST-fusion proteins in *E. coli*, purified on glutathione–Sepharose, and labelled with Alexa-488. Platelets were isolated from mouse blood by differential centrifugation and incubated with the α_M_I-domains for 30 min at room temperature. The platelets were then fixed with 2.5% glutaraldehyde in phosphate-buffered saline and analysed with a Facscan flow cytometer (Beckton-Dickenson), and mean fluorescence intensities were determined using the CellQuest software. Values for I-domain-free platelets were subtracted as the negative controls. Data are mean±s.d. (error bars) from two to three independent experiments, and each experiment was performed in triplicate.

Guided by the identification of the Mac-1-binding site for GPIbα, we took steps towards the generation of a mutant mouse with a double alanine substitution corresponding to the homologous murine sequence S^213^A and R^216^A (referred to as *muMac-1* mice; Supplementary Fig. 3). We first confirmed that the GPIbα specificity requirement of human α_M_I-domain also applied to purified murine α_M_I-domain. WT and mutant murine α_M_I-domains were expressed in *Escherichia coli* as gluthatione *S*-transferase (GST) fusion proteins and purified for use in ligand-binding assays. The *E. coli*-expressed α_M_I-domain has been used in multiple functional studies from our laboratories^45^,^48^ and from other investigators^49^. WT α_M_I-domain (S^213^ and R^216^), but not the S^213^A/R^216^A α_M_I-domain double mutant, bound to soluble GPIbα (Fig. 6a). Importantly, the binding of multiple other Mac-1 ligands, including ICAM-1, iC3b, CD40 ligand (CD40L) and fibrinogen, was unaffected by mutation of these two amino acids required for GPIbα binding (Fig. 6b), indicating that the murine S^213^A/R^216^A α_M_I-domain mutant functions similarly to human α_M_I-domain mutants (T^213^A/R^216^A) that we characterized previously^23^. We also characterized the binding of fluorescent-labelled WT α_M_I-domain (S^213^ and R^216^), single α_M_I-domain mutants (S^213^A or R^216^A) and double α_M_I-domain mutants (S^213^A/R^216^A) to mouse platelets that abundantly express GPIbα. WT α_M_I-domain (S^213^ and R^216^) bound to mouse platelets and this binding was reduced with both single and double α_M_I-domain mutants (Fig. 6c). The residual binding of the α_M_I-domain double mutant (S^213^A/R^216^A) to platelets may reflect its binding to other platelet surface ligands, such as JAM-3 (ref. 50) or heparin/heparan sulfate^51^.

**Figure 7:**
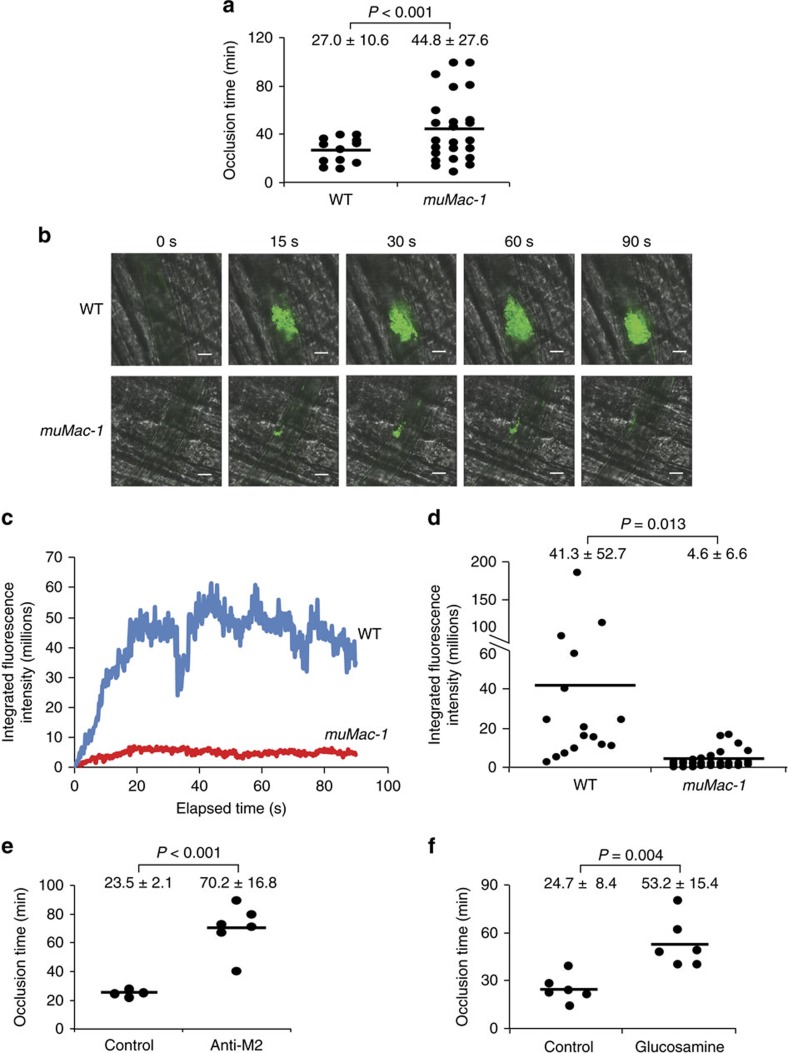
Mac-1 regulates thrombus formation through its interaction with platelet GPIbα. (**a**) Occlusion times in WT (*n*=12) and *muMac-1* (*n*=24) mice after carotid artery photochemical injury (mean±s.d.). Thrombus formation after laser-induced injury to the arteriolar wall of the cremaster microvasculature of *muMac-1* mice was compared with that of WT mice using intravital microscopy (**b**–**d**). Platelets were labelled *in vivo* using a fluorescein isothiocyanate-conjugated rat anti-mouse CD41 antibody. (**b**) Representative intravital images (*n*=16 for WT and 31 for *muMac-1*) at indicated times following laser pulse. Scale bar, 20 μm. (**c**) Continuous, real-time thrombosis profiles of arterioles from one representative experiment. (**d**) Integrated fluorescence intensity of platelets in individual arterioles over time (WT: *n*=16; *muMac-1*: *n*=31 arterioles; mean±s.d.). (**e**) Occlusion times in mice treated with anti-M2 antibody that disrupts Mac-1-GPIbα binding or IgG control (*n*=6 per group, mean±s.d.). (**f**) Occlusion times in mice treated with the small-molecule Mac-1-GPIbα inhibitor glucosamine or buffer control (*n*=6 per group, mean±s.d.). *P* values are obtained by unpaired two-tailed *t-*test.

To determine whether Mac-1 regulates thrombosis through its interaction with platelet GPIbα rather than other ligands, we subjected *muMac-1* mice to carotid artery photochemical injury (Fig. 7a). The mean time to occlusive thrombus formation was prolonged significantly in *muMac-1* (44.8±27.6 min) compared with WT mice (27.0±10.6 min). Importantly, we verified that complete blood count of whole blood from *muMac-1* mice was similar to that of WT mice (Supplementary Table 1), and that platelet activation in response to agonist stimulation (that is, expression of P-selectin and activated α_IIb_β_3_) was unaffected in platelets isolated from *muMac-1* compared to WT mice (Supplementary Fig. 4). Platelet adhesion (Supplementary Fig. 1g) and spreading (Supplementary Fig. 1h) on collagen were unimpaired in *muMac-1* mice. Next, we examined haemostasis in *muMac-1* mice. There was no difference in tail bleeding time between WT and *muMac-1* mice (mean bleeding time WT: 69±35 s versus *muMac-1*: 65±29 s, *n*=8–9 per group; Supplementary Fig. 5a). Finally, key leukocyte functions, including migration (as assessed by peritoneal macrophage accumulation after thioglycolate-induced peritonitis) and phagocytosis of Red Zymosan fluorescent beads, were unaffected in *muMac-1* mice (Supplementary Fig. 5b,c,d).

Thrombus formation after laser-induced injury to the arteriolar wall in the cremaster microcirculation of *muMac-1* mice was also compared with that of WT mice, using intravital microscopy^30^. In WT mice, platelet accumulation in arterioles was evident within 15 s of laser injury and increased progressively over the 90 s observation period; in contrast, platelet accumulation was markedly attenuated in *muMac-1* mice (Fig. 7b). Analysis of the growth curves of continuous, real-time thrombus profiles assessed by integrated fluorescence intensity of labelled platelets over time showed marked attenuation of platelet thrombus growth in *m*u*Mac-1* mice (WT: 41.3±52.7 × 10^6^, *n*=16 arterioles versus *muMac-1*: 4.6±6.6 × 10^6^, *n*=31 arterioles; Fig. 7c,d). Mean % inhibition over time was 89%. Taken together, these observations indicate that Mac-1-GPIbα regulates both large vessel and small vessel arterial thrombosis.

### Antibody targeting Mac-1:GPIbα inhibits thrombus formation

Having established a role for Mac-1 and its interaction with platelet GPIbα in thrombosis using genetic approaches, we next investigated the effect of antibody targeting of Mac-1:GPIbα interaction on thrombosis. We have reported previously on the generation of an antibody targeting α_M_I-domain P^201^–K^217^ (termed anti-M2) that selectively blocks the binding of Mac-1 to GPIbα, but not to other Mac-1 ligands^24^. This antibody attenuates inflammation and tissue injury in a variety of animal models, including restenosis^24^, vasculitis^25^, glomulerulonephritis^26^ and experimental autoimmune encephalomyelitis^27^. Anti-M2 (100 μg via tail vein) was injected into mice before photochemical carotid artery injury. Strikingly, antibody targeting of Mac-1:GPIbα prolonged significantly the time to occlusive thrombus formation to 70.2±16.8 min in anti-M2-treated mice compared to 23.5±2.1 min in control IgG-treated mice (Fig. 7e).

### Small-molecule screen for inhibitors of Mac-1:GPIbα binding

An antibody raised to the M2 peptide sequence in the α_M_I-domain of integrin blocks binding of GPIbα and platelets to the integrin and to leukocytes^24^. The anti-M2 antibody blocks Mac-1:GPIbα binding, but does not inhibit binding of other Mac-1 ligands. This behaviour suggests that small-molecule inhibitors of Mac-1:GPIbα might be developed for therapeutic utility. We have taken the initial steps to identify lead compounds with such blocking activity. We tested the 2,000 compounds in Spectrum collection from Microsource.

For initial screening of the library, we tested the compounds at a 100 μM concentration as inhibitors of binding recombinant fluorescently labelled α_M_I-domain to Chinese hamster ovary (CHO) cells expressing GPIbαβ. A representative screening assay showing the differential activity of 10 consecutive compounds tested, including both positive and negative hits, is shown in Supplementary Fig. 6a. Altogether, we obtained 97 positive hits, which inhibited the interaction by about 50% and then retested these at concentrations of 10, 30 and 50 μM. Eliminating those compounds that did not inhibit or were inhibitory at a single concentration reduced the number of compounds to 50. Of these, we moved forward with 36 compounds that were readily available.

Several approaches were developed to further characterize and select among the 36 positive hits. As negative selection criteria, we excluded compounds that caused clumping of neutrophils and platelets. As a specificity control, we excluded compounds that inhibited the adhesion of HEK-293 cells expressing Mac-1 to immobilized fibrinogen, another ligand of the integrin. As positive controls, we measured direct binding of the compounds to α_M_I-domain or to GPIbα in surface plasmon resonance (SPR) experiments. Compounds in both categories were identified, and several bound at low micromolar to sub-micromolar concentration.

Four more sets of assays have been performed subsequently: (1) SPR to determine which compounds interacted with the I-domains of four β_2_-integrins, α_L_β_2_, α_M_β_2_, α_X_β_2_ and α_D_β_2_; (2) inhibitory activities of the compounds on adhesion of HEK-293 cells expressing α_M_β_2_ to CHO cells expressing GPIbα; (3) effects of the compounds on the interaction of naturally occurring cells: inhibition of human platelet phagocytosis by human neutrophils; and (4) binding of labelled α_M_I-domain to human platelets was assessed, an assay that allowed us to assess the potency of the compounds as inhibitors of platelet–integrin interaction.

**Figure 8:**
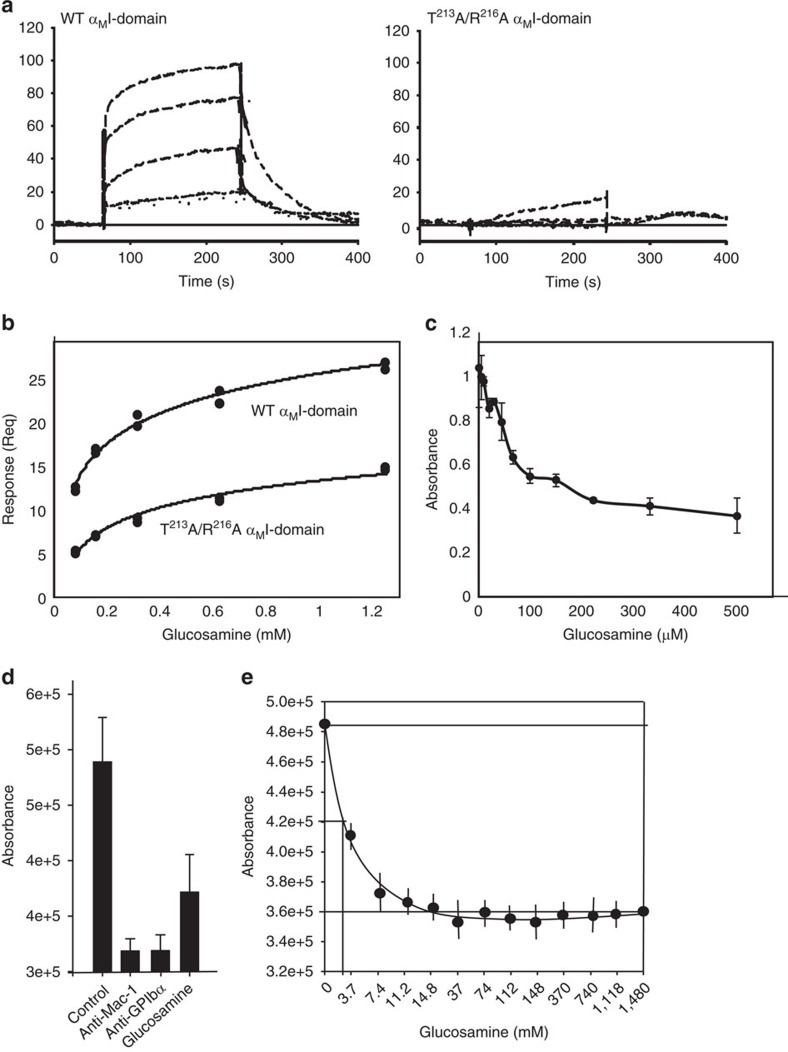
Glucosamine binds to the α_M_I-domain of Mac-1 and inhibits Mac-1 and GPIbα binding. (**a**) Binding of glucosamine to human WT and mutant (T^213^A/R^216^A) α_M_I-domain was assessed in real time by SPR. The α_M_I-domains were immobilized on CM5 biosensor chips at ∼2,500 RU, and SPR sensograms were obtained by injecting various concentrations of glucosamine over the immobilized α_M_I-domain proteins. (**b**) Steady-state binding isotherms for human WT and mutant (T^213^A/R^216^A) α_M_I-domain. Responses at the end of the association phase (Req) were used to determine *K*_0.5_ by fitting the curves to a steady-state affinity model (response units versus concentration). For this set of experiments, the α_M_I-domains were immobilized at ∼6,000 RU (in contrast to **a**). (**c**) The effect of glucosamine on binding of α_M_I-domain to purified GPIbα. Wells of Costar 96-well plates were coated with 1 μM GPIbα, post-coated with 0.5% PVP and 1 μM GST-tagged α_M_I-domain was added in the presence of glucosamine, 4–500 μM. After 15 min in room temperature, plates were washed and bound I-domain was quantified using anti-GST pAb and horseradish peroxidase (HRP)/3,3′,5,5′-tetramethylbenzidine system. Absorbance was read at 450 nm. (**d**) The effect of glucosamine on Mac-1:GPIbα binding was investigated using a cell-binding assay with Mac-1-transfected 293 and GPIbαβ-transfected CHO cells. Binding of transfected cells in presence of anti-Mac-1 (ICRF44), anti-GPIbα (VM16d) or glucosamine (50 μM). (**e**) Dose-dependent inhibition of Mac-1 293 cells binding to GPIbαβ-transfected CHO cells by glucosamine. Data are mean±s.d. (error bars) from two to three independent experiments with each experiment performed in triplicate.

On the basis of these assays, a group that bound specifically to the α_M_I-domain was identified. A representative of this group was glucosamine, which we explored in further *in vitro* and *in vivo* experiments. In SPR experiments, WT and α_M_I-double mutant were immobilized on CM5 chips, and binding of glucosamine to the surface was monitored in a BiaCore 3000 instrument. Under the conditions used, while glucosamine bound to WT α_M_I-domain (Fig. 8a, left) in a concentration-dependent manner, no consistent interaction of the sugar derivative with the α_M_I-double mutant was detected (Fig. 8a, right). Because of the low signal obtained not only with the α_M_I-double mutant but also with the WT α_M_I-domain, we did not attempt to derive binding constants from these data. The literature indicates that evaluation of carbohydrate binding by SPR can be challenging^52^. Hence, we increased the coating density of the WT and α_M_I-double mutant on the chip from ∼2,500 relative units (RU) on the chip to ∼6,000 RU on the CM5 chip surface. Under this condition, we detected low-level binding of both WT α_M_I-domain and the α_M_I-domain double mutant (Supplementary Fig. 7) although the responses obtained with WT α_M_I-domain was consistently greater. In attempt to derive a binding constant, we used a steady-state approach to interpret the data (Fig. 8b), an approach found to be useful for analysis of progress curves from low-affinity interactions^52^. The binding isotherm for WT α_M_I-domain yielded an estimated Kd of ∼100 μM, whereas that of the α_M_I-double mutant was ∼244 μM. This difference indicates that one site of glucosamine binding is influenced by S^213^A/R^216^A mutation. Independent approaches were implemented to verify that glucosamine inhibits the interaction of α_M_I-domain with GPIbα. First, binding of biotinylated GPIbα to α_M_I-domain immobilized on microtitre plates was detected with streptavidin–horseradish peroxidase (HRP) conjugate. This interaction was inhibited in a dose-dependent manner by glucosamine (Fig. 8c). The concentration of glucosamine inhibiting binding by 50% (IC50) was ∼50 μM, approximating the Kd estimated from the SPR experiments. Second, an assay in which binding of Alexa-488-labelled Mac-1-expressing 293 cells to adherent GPIbα-transfected CHO cells was developed. This interaction was inhibited by an anti-Mac-1 antibody, by an anti-GPIbα antibody or by glucosamine (Fig. 8d). Indeed, in this assay, glucosamine produced dose-dependent inhibition (Fig. 8e). We also compared the specificity of glucosamine to several similar carbohydrate derivatives (Supplementary Fig. 6b). Of these compounds, only glucosamine produced significant inhibition of GPIbα binding to the α_M_I-domain (Supplementary Fig. 6b).

**Figure 9:**
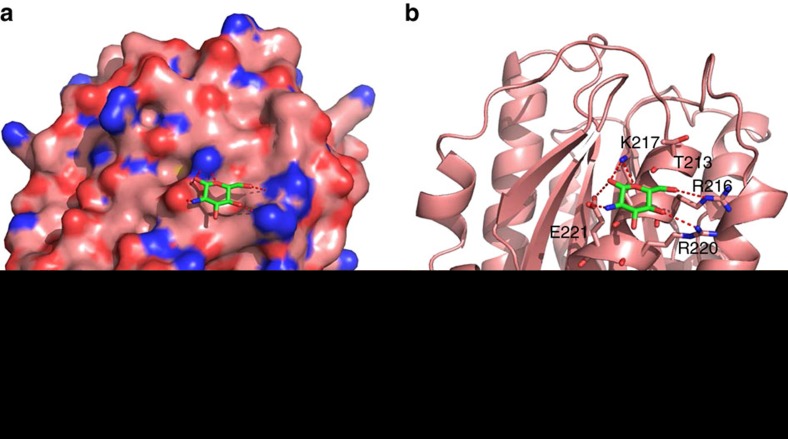
Binding mode of glucosamine to α_M_I-domain of Mac-1. (**a**) Glucosamine binds to a small pocket on α_M_I-domain of Mac-1 (shown in surface presentation). (**b**) Key residues of the α_M_I-domain (cartoon presentation) potentially involved in the interaction with glucosamine (shown in stick mode). Potential H-bonds formed between α_M_I-domain and glucosamine are displayed by red dashed lines.

To better understand how glucosamine interacts with α_M_I-domain, we performed docking analysis to calculate the potential binding mode of glucosamine by using Schrödinger Maestro software. Multiple possible conformations were generated by defining R^216^ as potential binding site. One conformation with best docking score of ‘−4.053’ was selected. In this binding mode, glucosamine binds to a small pocket of α_M_I-domain formed by the α-helix residues from T^211^ to E^221^ (Fig. 9a). It is spatially close to T^213^ and forms several critical H-bonds with the side chains of R^216^, K^217^, R^220^ and E^221^ (Fig. 9b). This docking mode provides a basis for understanding our mutagenesis study that showed that T^213^A/R^216^A mutant abolished the glucosamine binding. In comparing the conformation of glucosamine docked in this model, the root mean squared deviations in the eight next best fits were all quite similar, varying from −0.026 to −0.093, to that shown in Fig. 9a.

The anti-thrombotic potential of glucosamine as an inhibitor of Mac-1:GPIbα binding was then investigated in the carotid artery photochemical injury model. Glucosamine (27 μg) or phosphate-buffered saline vehicle control were administered via tail vein injection immediately before carotid artery injury. The small-molecule Mac-1:GPIbα inhibitor glucosamine significantly prolonged thrombus formation to 53.2±15.4 min compared to 24.7±8.4 min for vehicle control (Fig. 7f). We also examined haemostasis in mice treated with glucosamine. There was no difference in tail bleeding time between mice treated with buffer or glucosamine (mean bleeding time buffer: 73±31 s versus glucosamine: 82±35 s, *n*=12 per group; Supplementary Fig. 5e). Taken together, these observations indicate that genetic, antibody and small-molecule targeting of Mac-1:GPIbα binding are capable of influencing arterial thrombus formation *in vivo*.

## Discussion

In this study, we have identified a new pathway of thrombosis involving leukocyte Mac-1 and platelet GPIbα, and interfering with this pathway does not affect parameters of haemostasis. This conclusion is supported by the following data: (1) mice with deficiency of Mac-1 (*Mac-1*^*−/−*^) or mutation of the Mac-1-binding site for GPIbα (*muMac-1*) have delayed thrombus formation after injury to large and small arteries; (2) platelet count, platelet activation, plasma coagulation activity and bleeding time were similar in WT and *Mac-1*^*−/−*^ mice; (3) adoptive leukocyte transfer rescued defective thrombus formation in *Mac-1*^*−/−*^ mice; (4) Mac-1-GPIbα induces ‘outside-in’ Mac-1 signalling (that is, phosphorylation of PKC and downregulation of Foxp1), and Mac-1-dependent regulation of the transcription factor Foxp1, which regulates TF expression, contributed to the thrombosis defect as evidenced by prolonged thrombotic occlusion time in *macFoxp1tg* mice; and (5) antibody and small-molecule targeting of Mac-1:GPIbα inhibited thrombus formation.

Platelet–leukocyte interactions bidirectionally induce signals that amplify pro-inflammatory and pro-thrombotic cellular responses^12^. One of the key implications of our findings is the central importance of leukocyte Mac-1 and platelet GPIbα for leukocyte–platelet interactions *in vivo*. Other reported interactions contributing to Mac-1-independent leukocyte–platelet conjugate formation include thrombospondins bridging between GP IV receptors on platelets and monocytes^53^, and P-selectin on activated platelets binding with leukocyte P-selectin glycoprotein ligand-1 (refs 54, 55). However, stable accumulation of leukocytes to adherent platelets *ex vivo* under experimental conditions of arterial shear or to the endothelial–denuded vessel lined with platelets *in vivo*, requires leukocyte Mac-1 and platelet GPIbα (ref. 24). Other potential Mac-1 ligands present on the platelet membrane include fibrinogen (bound to α_IIb_β_3_)^56^,^57^, ICAM-2 (ref. 58) and JAM-3 (ref. 50). However, a leukocyte–platelet interaction mediated by fibrinogen bridging between Mac-1 and α_IIb_β_3_ has been largely discounted by Ostrovsky *et al*.^59^ who found that neither RGDS peptides nor the replacement of normal platelets with thrombasthenic platelets (that is, lacking α_IIb_β_3_) affected the accumulation of the leukocytes on platelets. Although Mac-1 binds ICAM-1, this receptor is not found on platelets. Platelets express a related receptor, ICAM-2 (ref. 58), but Diacovo *et al*.^16^ have shown that ICAM-2 blockade has no effect on the firm adhesion of neutrophils on monolayers of activated platelets under flow. Santoso *et al*.^50^ have reported that Mac-1 may also bind to platelet JAM-3, cooperating with GPIbα to mediate neutrophil–platelet adhesive contacts *in vitro*. However, we have reported previously that anti-M2, which is capable of blocking platelet-dependent leukocyte recruitment both *ex vivo* and *in vivo*, had minimal inhibitory effect on Mac-1-dependent adhesion to JAM-3 (ref. 24).

Genetic (*muMac-1* mice) and antibody approaches were utilized to investigate the importance of Mac-1 binding to GPIbα, but not to other Mac-1 ligands in thrombosis. The relative specificity of anti-M2 inhibitory action towards GPIbα (that is, non-inhibitory towards ICAM-1, fibrinogen, JAM-3 and C3bi) suggests a minor contribution of other ligands for Mac-1 in the context of thrombosis.

Previous work from our laboratories has demonstrated that leukocyte engagement of platelet GPIbα via Mac-1 induces platelet ‘outside-in’ signalling and platelet activation^31^. We now provide new evidence that engagement of platelet GPIbα via Mac-1 induces ‘outside-in’ Mac-1 signalling that leads to phosphorylation of PKC delta and downregulation of Foxp1 in monocytic cells. Thus, blockade of the initial cell–cell conjugation mediated by Mac-1:GPIbα may prevent bidirectional signalling that amplify thrombus formation, and account for the effectiveness of Mac-1:GPIbα inhibition in reducing thrombus formation *in vivo*.

Having established that adoptive transfer of WT neutrophils or PBMCs partially corrects the thrombosis defect in *Mac-1*^*−/−*^ mice, one needs to consider the possibility that there may be distinct mechanisms driving neutrophil versus mononuclear leukocytes in thrombus formation. While TF may be important in mononuclear cell-dependent thrombosis, it is controversial whether neutrophils express TF. Neutrophils are known to regulate thrombosis through the formation of neutrophil extracellular traps (NETs), which stimulate thrombus formation and coagulation and are abundant in thrombi in animal models of deep vein thrombosis^60^. The molecular basis of NET generation (known as NETosis) is a complex process requiring reactive oxygen species production^61^ and neutrophil proteases (that is, neutrophil elastase, myeloperoxidase and peptidylargine deiminase-4)^62^. Highly relevant to the present study, Neeli *et al*.^63^ have provided evidence that the Mac-1 itself may be involved in the initiation of changes in the neutrophil cytoskeleton that facilitate the breakdown of nuclear and plasma membranes for the releases of NETs.

The present observations suggest a possible target for therapeutic intervention in cardiovascular and thrombotic diseases. In particular, the specificity of antibody or small-molecule inhibitory action towards Mac-1:GPIbα suggests that it might be possible to inhibit pro-thrombotic leukocyte–platelet interactions without affecting other Mac-1 functions. Our study identifies glucosamine as one such small-molecule antagonist. The failure of other molecules of similar size and composition to glucosamine to block α_M_I-domain:GPIbα interaction augers well for the possibility of detailed structure–activity analyses to identify more potent small-molecule antagonists. Our modelling study (Fig. 9) suggests that several closely related binding modes would allow glucosamine to bind in close proximity to T^213^/R^216^ and interfere with GPIbα binding. Ultimately, solving the crystal structure of glucosamine or other small molecules identified in our screen or of an anti-M2 bound to the α_M_I-domain may lead to a new class of anti-thrombotic therapy. At a more fundamental level, the results of this study suggest that thrombosis and haemorrhage may be uncoupled at the level of the Mac-1:GPIbα interaction. In particular, the specificity of antibody or small-molecule inhibitory action towards Mac-1:GPIbα suggests that it might be possible to inhibit pro-thrombotic leukocyte–platelet interactions without affecting other Mac-1 functions. Deficiency of Mac-1 did not interfere with tail bleeding time, platelet activation and plasma coagulation activity (that is, aPTT and thrombin generation). The identification of a new platelet-dependent pathway of thrombosis that does not affect haemostatic parameters, such as bleeding time and platelet adhesion and spreading, has possible clinical implications. Thrombotic cardiovascular diseases, including myocardial infarction and stroke, are the leading cause of death in developed countries^1^. Total US healthcare expenditures in 2009 for coronary heart disease and stroke were a staggering $165.4 billion and $68.9 billion, respectively^1^ with pharmacologic therapies estimated to exceed $20 billion worldwide^1^. Antiplatelet agents and anticoagulants are used in the treatment of acute coronary syndrome and in primary and secondary prevention of coronary artery disease and stroke^64^,^65^. Current drugs are subject to significant bleeding risk, which is associated with increased mortality^2^,^3^,^4^. While new antiplatelet (for example, prasugrel, ticagrelor and vorapaxar) and anticoagulant (for example, apixaban, dabigatran and rivaroxaban) agents have been approved on the basis of superior efficacy or other clinical advantages (for example, fixed dosing without the need for monitoring), these therapeutic advances are associated with a 25–30% increase in the rate of bleeding or transfusion. There is emerging experimental evidence distinguishing the molecular and cellular mechanisms of haemostasis and thrombosis^5^,^6^. The interaction between leukocyte Mac-1 and platelet GPIbα is now positioned as a novel and targetable mediator of thrombosis, but not haemostasis (that is, reduced bleeding risk).

## Methods

### Materials

Antibody to mouse P-selectin/CD62P conjugated to fluorescein isothiocyanate (Wug.E9) and antibody to mouse activated integrin α_IIb_β_3_ (GPIIb/IIIa) conjugated to R-phycoerythrin (JON/A) were purchased from emfret Analytics (Würzburg, Germany, Catalogue #: D200). Polyclonal antibody (termed anti-M2) to the Mac-1-binding site for GPIbα was generated by YenZym Antibodies, LLC (South San Francisco, CA). Antibody to TF was purchased from R&D Systems Inc. (AF3178, Minneapolis, MN). Rose Bengal (4, 5, 6, 7-tetrachloro-3′, 6-dihydroxy-2, 4, 5, 7-tetraiodospiro (isobenzofuran-1(3H), 9 [9H] xanthan)-3-1 dipotassium salt) was purchased from Sigma-Aldrich (St Louis, MO). Human α-thrombin was purchased from Haematological Technologies (Essex Junction, VT, Catalogue # HCT-0020). Collagen (Catalogue # 101562) and arachidonic acid (Cat # 101297) were purchased from Bio/DATA Corporation (Horsham, PA).

### Mice

All mice had a congenic C57BL/6 background and were maintained in animal facilities at Case Western Reserve University School of Medicine.

*Mac-1*^*−/−*^ mice were generated in the laboratory of Dr Christie Ballantyne^39^. Mutant Mac-1 mice expressing the S^213^A/R^216^A double-mutant α_M_I-domain were generated at Ozgene Pty. Ltd (Bentley, Australia) using Cre-mediated inversion strategy by flanking an inverted exon 7 fragment, containing the knock-in sequence with lox66 and lox71 sites^66^,^67^. The following four fragments were generated by PCR from C57BL/6 genomic DNA and cloned into the Ozgene plasmid PacF 10000113-A08: (a) mutant exon 7 fragment (KI)—the KI fragment contained PacI and ClaI sites for cloning, an EcoRV site for genomic screening and a lox71 site at the 3′-end; (b) WT exon 7 fragment (wt)—the wt fragment contained ClaI and AscI sites for cloning and a lox66 site at the 5′-end; (c) 5′-homology arm (5H)—the 5H arm contained AscI and AatII sites for cloning, and NdeI and MfeI sites for genomic screening; and (d) 3′-homology arm (H3)—the H3 arm contained an AatII site for cloning and an NdeI site for genomic screening. The targeting vector containing all four fragments was sequenced and introduced into embryonic stem cells by electroporation. Embryonic stem cells in which homologous recombination occurred as detected by Southern blot analysis were injected into a mouse blastocyst to generate chimeric mice. Offspring of chimera × C57Bl/6 mating confirmed as wt/flox underwent three additional breeding to remove Neo, FLP and Cre cassettes, and to bring the mutated exon 7 into reading frame. Breeding step 1: to remove the selection cassette from a heterozygous wt/flox line by breeding to heterozygous wt/FLP mice. Breeding step 2: to remove the FLP gene and to generate a global Cre inverted wt/KI line by breeding the wt/floxΔneo–wt/FLP line generated in breeding step1, to homozygous Cre mice. Breeding step 3: to remove the Cre gene by breeding the wt/KI—wt/Cre mice generated in step 2, back to C57BL/6J mice. After the establishment of the mutant mouse colony, mice were routinely genotyped by PCR of genomic tail DNA using the following primers in two separate reactions to generate a mutant band of 469 bp (YM121 and YM131) and a WT band of 585 bp (YM121 and YM128): YM121 (5′-GTCCTACCTCGACATGTTCTTTTC-3′); YM128 (5′-AGCATAGGCTTAATCCACCTCTCT-3′); and YM131 (5′-ACACTACTTTGGCGATCCCGGC-3′). *macFoxp1tg* mice were generated in our laboratory as reported^34^.

### Platelet isolation

Mouse platelets were isolated from the whole blood obtained by terminal inferior vena cava phlebotomy as described^10^,^68^. Briefly, platelet-rich plasma was prepared by centrifugation, and platelets were suspended in Tyrode’s buffer. Platelet suspensions were adjusted to final density after counting particles >3 fl using a Z1 series Coulter Counter (Beckman Coulter, Fullerton, CA) equipped with a 50 μm aperture or were measured as part of a complete blood count of sodium citrate-anticoagulated mouse blood on a HEMAVET 950FS system in the Case Comprehensive Cancer Center, Case Western Reserve University School of Medicine.

### Activated partial thromboplastin time

The aPTT was performed using Amelung KC4 coagulation analyser (Sigma, St Louis, MO), as described previously^10^,^69^. Briefly, 100 μl of sodium citrate-anticoagulated plasma was incubated with 50 μl of aPTT reagent (Siemens, Washington DC) at 37 °C for 5 min. A volume of 50 μl of 30 mM calcium chloride was then added and the time to clot formation was recorded.

### Thrombin generation

TF-induced thrombin generation time was performed, as previously described^10^,^70^. Briefly, a 1:2 dilution of mouse plasma was incubated with ∼3 pM TF (3 μl of 1:60 dilution of stock Innovin, Siemens) and 0.42 mM Z-Gly-Gly-Arg-AMC. The reaction was initiated with the injection of 0.16 M calcium chloride, final concentration 16 mM. Substrate hydrolysis was measured on a fluorescent plate reader (NOVOstar, BMG Labtech). The thrombin generation data are expressed as an arbitrary rate of fluorescent accumulation as determined by the second derivative of the raw fluorescent values. The lag time, peak height and total area under the curve were calculated using Prism software (Graphpad, San Diego, CA).

### Platelet α-granule release and GPIIb/IIIa activation

Platelet activation assay was performed as previously described^10^. Briefly, mouse platelets were stimulated for 10 min at room temperature, with agonists. The Wug.E9 and JON/A antibodies were added to detect the expression of P-selectin (CD62P) and activated α_IIb_β_3_ (GPIIb/IIIa), respectively, following the instructions of the manufacturer. After 20 min, platelets were fixed for fluorescence-activated cell sorting (FACS) analysis by addition of formaldehyde. Platelets were distinguished on the basis of side- and forward-light scatter, and the mean fluorescence intensity of platelets was measured using FACSDiva LSRII (Becton Dickinson) and analysed using FACS Diva 6.2 or FlowJo v10.

### Photochemical carotid artery thrombosis

This thrombosis model was performed, as previously described^10^. Briefly, 7- to 9-week-old male mice were anaesthetized and placed on a dissecting microscope (Leica S4E, Leica Microsystems, IL, USA). A midline surgical incision was made to expose the right common carotid artery, and a Doppler flow probe (MC 0.5PSL Nanoprobe, Model 0.5 VB, Transonic Systems, Ithaca, NY) was placed under the vessel. The probe was connected to a flowmeter (Transonic Systems Model TS420) and was interpreted with a computerized data acquisition programme (Windaq, DATAQ Instruments, Arkron, OH). Rose Bengal was injected into the tail vein to administer a dose of 50 mg kg^−1^ (refs 28, 29). The mid portion of the common carotid artery was then illuminated with a 1.5 mW green light laser source (540 nm; Melles Griot, Carlsbad, CA) 5 cm from the artery. Blood flow was monitored continuously from the onset of injury. The time to occlusion, determined only after the vessel remained closed with a cessation of blood flow for 10 min, was recorded. In a separate group of animals, 100 μg of anti-M2 antibody or 27 μg of glucosamine was also infused into mice via tail vein injection to determine the effect of these GPIbα- and Mac-1-blocking reagents on thrombus formation.

### Laser injury to microcirculation using intravital microscopy

Thrombosis was induced, as previously described in male mice aged 11–12 weeks^10^,^30^. Briefly, thrombus formation *in vivo* after laser-induced injury to the arteriolar wall in the cremaster microcirculation of WT was compared with that of *Mac-1*^*−/−*^ mice using intravital microscopy (VIVO, 3I Inc.) performed as described previously^30^. Platelets were labelled *in vivo* using a fluorescein isothiocyanate--conjugated rat anti-mouse CD41 antibody (BD Pharmingen, San Jose, CA) at a dose of 0.4 μg per g body weight of mouse.

### Adoptive transfer experiments

Blood from the inferior vena cava of two mice was collected directly into 3.8% sodium citrate (9:1 blood:citrate) and diluted with equal amount of Tyrode’s buffer. PBMCs were isolated using histopaque (Catalogue #: Histopaque-1077, Sigma-Aldrich, St Louis, MO, USA). Wright-Giemsa staining showed that monocytes represented up to 70% of the PBMC population. Neutrophils were isolated from anticoagulated and diluted blood using Percoll (P1644, Sigma-Aldrich) density gradient centrifugation method. Wright-Giemsa staining showed >85% purity of neutrophils.

### Mouse bleeding times

Tail bleeding times were measured by transecting the tails of sex (male and female)- and age (2–3 months old)-matched anaesthetized mice 5 mm from the tip, as previously described^10^,^69^. The transected tail tip was placed into a beaker containing saline at 37 °C and the time to complete cessation of bleeding for 30 s and 3 min was determined with a stopwatch. Alternatively, the transected tail tip was blotted with filter paper every 15 s and the time to complete cessation of bleeding was determined with a stopwatch.

### TF reporter assay

NIH/3T3 cells were co-transfected with 50 ng of pCMV-β-gal, 0.5 μg of human TF promoter-luciferase and 3.0 μg of pcDNA3.1 vector or pcDNA3.1/FOXP1 construct DNA by Lipofectamine 3000 transfection reagent (Thermo Fisher Scientific, Waltham, MA). Two days after transfection, some samples were treated with 20 nM phorbol 12-myristate 13-acetate for 16 h. Cells were then lysed by 1 × reporter buffer (Promega, Madison, WI) and measured for firefly luciferase activity using Luciferase Assay System reagents (Promega) in a GloMax Microplate Luminometer (Promega). β-galactosidase activity was measured by Enhanced Beta-gal Assay Kit (Genlantis, San Diego, CA). Duplicate measurements of triplicate wells for each sample were performed and the TF promoter-luciferase activities were normalized by the activity of the β-galactosidase internal control.

### Mac-1 clustering and signalling

Mac-1-expressing THP-1 monocytic cells were pre-treated with transforming growth factor-β1 (1 ng ml^*−*1^) and 1,25-(OH)_2_ vitamin D3 (50 nM) overnight at 37 °C. Six-well plates were coated with full-length soluble GPIbα (R&D) or N-terminal GPIbα (GC300) for 2 h at room temperature, then blocked by 0.1% polyvinylpyrrolidone (PVP, Sigma) at 37 °C for 1 h. Wells coated with PVP alone were used as control. THP-1 cells were treated with the β_2_-integrin-activating monoclonal antibody (mAb) KIM185 (10 μg ml^*−*1^) and then added to wells coated with full-length GPIbα or N-terminal GPIbα (GC300) to induce Mac-1 clustering and adhesion. After incubation for 2 h at 37 °C, adherent cells (Mac-1-clustered) or non-adherent cells exposed to PVP control wells (non-clustered) were collected, washed and lysed in 1 × RIPA buffer. Protein concentrations were determined by BCA assay, and protein samples (20 μg per lane) were resolved on 4–12% NuPAGE gel for western blot by anti-Phospho-PKC delta (tyr311) antibody (10 μl per 10 ml reaction, Catalogue #: 2055, Cell Signaling Technology). The membrane was re-blotted using anti-total PKC delta (10 μl per 10 ml reaction, Catalogue # ab182126, Abcam) and anti-β-actin (1 μg ml^−1^, Catalogue # A1978, Sigma-Aldrich) antibodies. Bands were visualized with HRP-conjugated secondary antibody followed by the enhanced chemiluminescence western blotting detection system (PerkinElmer Life and Analytical Sciences, Waltham, MA). For experiments evaluating the effect of Mac-1 clustering on the expression of Foxp1, THP-1 cells were added to wells pre-coated with 0.1% gelatin and gel-filtered human platelets or gelatin-coated wells alone overnight at 37 °C. Cells were then collected as above for immunoblotting with anti-Foxp1 antibody (1 μg ml^−1^, customer-designed polyclonal antibody raised against CDHDRDYEDEPVNEDME by Zymed Laboratories Inc., South San Francisco, CA) as described previously^34^.

### Thioglycolate-induced peritonitis and isolation of macrophages

Peritonitis was induced in 12- to 20-week-old mice by intraperitoneal injection of 1 ml sterile 3% (wt/vol.) thioglycolate broth. At 48–72 h, peritoneal cavities were lavaged by injecting 10 ml sterile PBS buffer twice with gentle abdominal massage. Leukocytes in the lavaged fluid were counted and macrophages were enriched after brief adhesion to tissue culture plastic to remove non-adherent lymphocytes, resulting in >95% macrophages.

### Phagocytosis assay

Live functional (adherent) peritoneal macrophages collected using the above method were aliquoted onto two 96-well plates for phagocytosis assay. Macrophages in one 96-well plate were stimulated by lipopolysaccharide (10 μg ml^−1^) for 24 h, followed by incubation with sonicated pHrodo Red Zymosan, a bioparticle fluorescent beads (from ThermoFisher Scientific), for 2 h at 37 °C. Cells on another plate were stained by BCECF to count seeded/treated live cells for further calibration of phagocytic cell number. The fluorescence intensity of pHrodo beads and BCECF-treated cells were recorded by fluorescent plate reader (CytoFluor II, PerSeptiveBiosystems). Images were captured by EVOS FL (LifeTechnologies).

### Expression of recombinant mouse α_M_I-domains

Mouse α_M_I-domain was expressed as a GST fusion protein. The coding region of the mouse I-domain (residues E^132^–A^318^) was PCR-amplified using the cDNA of murine α_M_ as a template, and the product was cloned into the pGEX-4T-1 expression vector (GE Healthcare Life Sciences, Little Chalfont, UK). The DNA sequence was verified, and the plasmid was used to transform *E. coli* strain BL-21 competent cells. Expression was induced with 5 mM isopropyl-1-thio-β-D-galactopyranoside (Sigma-Aldrich) for 3–5 h at 37 °C. The fusion protein was purified from the *E. coli* lysate by affinity chromatography on glutathione–Sepharose (GE Healthcare Life Sciences).

To mutate the residues corresponding to S^213^ (corresponds to T^213^ in human α_M_) and R^216^ to alanines, site-directed mutagenesis was performed by using the QuikChange II XL Site-Directed Mutagenesis Kit (Agilent Technologies, Santa Clara, CA) according to the manufacturer’s protocol. The pGEX-4T-1 construct containing WT mouse I-domain DNA was modified by PCR using two 52-mer mutagenic primers: 5′-GAATGGGAGGACAAAAACTGCCGCCGGGATCGCAAAAGTAGTGAGAGAACTG-3′ (forward) and 5′-CAGTTCTCTCACTACTTTTGCGATCCCGGCGGCAGTTTTTGTCCTCCCATTC-3′ (reverse). The product was treated with Dpn I endonuclease to digest the parental DNA template, and nicked vector was then transformed into the Gold Ultracompetent XL cells (Agilent) of the *E. coli* BL-21 strain. Individual bacterial clones were analysed by sequencing, and the presence of the desired mutations and the absence of other mutations in the cloned cDNA were verified. Expression and purification of the mutant I-domains as a GST-fusion protein followed the same procedure as for WT α_M_I-domain.

### Expression of recombinant soluble GPIbα

cDNA encoding amino acids 1–300 of GPIbα was amplified by PCR and cloned into the NheI and XhoI sites of pBIG-4f (ref. 71). CHO Tet-On cells (Clontech, Mountain View, CA) secreting the recombinant GPIbα-300 peptide were incubated for 48 h in serum-free medium (EX-CELL 302; Sigma) containing 10 mM biotin (Sigma) and 2 mg ml^−1^ doxycycline (Sigma). Conditioned medium was concentrated and desalted to remove free biotin using a PD-10 column (Sigma).

### Solid phase assays to test specificity and screen inhibitors

#### Ligand specificity of WT and mutant murine α_M_I domains

Wells of Costar 96-well HB plates were coated with 200 μl of 2 μg ml^*−*1^ soluble GPIbα, CD40L, iC3b and ICAM-1 (all R&D Systems, Minneapolis, MN), or 1 μg ml^*−*1^ human fibrinogen (Enzyme Research Laboratories, South Bend, IN) at 4 °C overnight and 1 h at 37 °C. DH fragment (molecular weight 100 kDa) was prepared by digestion of human fibrinogen with plasmin (Sigma-Aldrich) followed by ion-exchange chromatography on CM-Sephadex and by gel filtration on Sephacryl S-200 (both from Sigma), as described^72^. Unlike fibrinogen, DH fragment contains an exposed α_M_β_2_ recognition site and supports α_M_β_2_-dependent binding in a dose-dependent manner^46^. The wells then were post-coated 1 h at 22 °C with 300 μl 0.5% PVP (Sigma-Aldrich). GST-tagged α_M_I-domains were diluted with Hank’s balanced salt solution (HBSS) containing 20 mM HEPES (pH 7.4), 2 mM CaCl_2_ and 2 mM MgCl_2_ (HBSS/HEPES) to 5 μg ml^*−*1^ and added to the wells in 200 μl aliquots. As background controls, wells were coated with PVP only. After 1 h incubation at 37 °C, plates were washed with HBSS/HEPES and 200 μl of 5 μg ml^−1^ anti-GST antibodies conjugated to HRP in HBSS/HEPES, containing 0.5% BSA were added. After 1 h at 37 °C, the plates were washed with HBSS/HEPES, containing 0.1% Tween 80, and 200 μl HRP liquid substrate (Sigma-Aldrich) was added. After 5 min, the reaction was stopped by addition of 50 μl 1 M H_2_SO_4_ and absorbance at 450 nm was measured in a multiwell plate reader (Molecular Probes).

#### Inhibition assays

Several different variations of microtitre plate assays (96-well TC Costar plates) were used to test inhibitors of α_M_β_2_:GPIbα interaction. In one variation, plates were coated with 150 μl α_M_I-domain (1 μg ml^*−*1^) overnight 4 °C+1 h 37 °C. Plates were post-coated with 200 μl BSA (5 mg ml^*−*1^) for 1 h at room temperature and then washed with 150 μl HBSS, containing 20 mM HEPES (pH 7.2), 2 mM CaCl_2_ and 2 mM MgCl_2_ (HBSS/HEPES). Anti-α_M_ mAb M1/70, anti-M2 peptide, glucosamine or related carbohydrates at the indicated concentrations were added. Plates were incubated for 20 min at 22 °C, and then 3 μl of GPIbα–biotin conjugate (0.25 mg ml^*−*1^) were added. After 30 min at 37 °C, the plates were washed with HBSS/HEPES, and 150 μl streptavidin–HRP conjugate was added. After 30 min at 37 °C the plates were washed with HBSS/HEPES. Bound GPIbα was detected using 3,3′,5,5′-tetramethylbenzidine liquid substrate (Sigma), and absorbance was measured at 450 nm.

In another variation, Mac-1-expressing HEK293 cells^73^ and cells co-expressing the cDNAs for GPIbα and GPIbβ in CHO cells were prepared as described^74^. Binding of Alexa-488-labelled Mac-1 cells to GPIbα/β cells grown in microtitre plate wells was assessed in the presence of anti-Mac-1 (ICRF44), anti-GPIbα (VM16d) or glucosamine. After 30 min at 37 °C, the plates were washed a minimum of three times and absorbance was read in a microtitre plate reader. A third assay was used to screen the 2,000 small molecules within the Spectrum collection from Microsource. GPIbα/β cells were grown to confluence in microtitre plates. After washing, Alexa-488-labelled α_M_I-domain (1 μg ml^*−*1^) was added together with 100 μM (final concentration) the test inhibitor in HBSS containing 10 μg ml^*−*1^ BSA in a total volume of 200 μl aliquots. As background control, wells coated with PVP only. After 30 min at 37 °C, plates were washed with HBSS/HEPES and read in a spectrophotometer; background binding was taken as the absorbance in the presence of mAb (ICRF44). Each test compound was tested in triplicate, and eight wells on each plate were used to determine background, nonspecific binding.

### Surface plasmon resonance

Binding of glucosamine to human WT and mutant α_M_I-domains was assessed in real time by SPR using a Biacore3000 instrument (Biacore, Uppsala, Sweden). The α_M_I-domains were immobilized on CM5 biosensor chips using the standard amine coupling chemistry according to the manufacturer’s instructions to achieve either a typical (2,500 RU) or higher coating density (6,000 RU). Experiments were performed at room temperature in 10 mM HEPES (pH 7.4) containing 150 mM NaCl and 0.005% surfactant P20 at a flow rate of 25 μl min^−1^. SPR sensograms were obtained by injecting various concentrations of glucosamine over the immobilized α_M_I-domain proteins. The chip surfaces were regenerated by injecting a short pulse of 5 mM NaOH. The curves of buffer only and the blank flow cell were subtracted from the binding curves, and the resulting progress curves were analysed in overlay plots using BIAevaluation software (version 4.01, GE Healthcare) or a steady-state approach.

### Molecular docking of glucosamine on the α_M_I-domain

Molecular docking was performed using Maestro 9.9 software (Schrödinger Release 2014-3, Schrödinger, LLC) with the following procedures.

#### Ligand preparation

The structure of D-glucosamine in structure data file (SDF) format was downloaded from PubChem (PubChem CID:439213). It was later processed using ‘LigPrep’ module in Maestro. The compound was desalted, and possible ionization states in the pH range of 5.0–9.0 and tautomers were generated by Epik mode in the force field of OPLS_2005.

#### Protein preparation

The coordinates of α_M_I-domain was derived from the Protein Data Bank (PDB code: 1BHO). Before docking, the protein was processed and refined with ‘Protein Preparation’ module in Maestro.

#### Docking grid generation

The residue R^216^ of α_M_I-domain was used to define the centre of the docking pocket; the scaling factor and the partial charge cut-off of the van der Waals radii were set to 1.0 and 0.25, respectively. This step was done by using ‘Receptor Grid Generation’ module in Maestro.

#### Glide docking

Prepared glucosamine was docked into the defined binding pocket using SP (standard-precision) and XP (extra-precision) docking modes, respectively. The docking conformation analysis was performed using PyMol (Version 1.3, Schrödinger, LLC).

### Statistical analysis

Data are presented as mean±s.d. Comparisons between groups were performed by unpaired, two-tailed Student’s *t-*test. Probability values <0.05 were considered significant.

### Study approval

Animal care and procedures were reviewed and approved by the Case Western Reserve University School of Medicine Institutional Animal Care and Use Committees and performed in accordance with the guidelines of the American Association for Accreditation of Laboratory Animal Care and the National Institutes of Health.

### Data availability

The data supporting the findings of the current study are available from the corresponding author on reasonable request.

## Additional information

**How to cite this article:** Wang, Y. *et al*. Leukocyte integrin Mac-1 regulates thrombosis via interaction with platelet GPIbα. *Nat. Commun.* **8,** 15559 doi: 10.1038/ncomms15559 (2017).

**Publisher’s note:** Springer Nature remains neutral with regard to jurisdictional claims in published maps and institutional affiliations.

## Supplementary Material

Supplementary Information Supplementary Figures and Supplementary Table.
