# Corrigendum: Leukocyte integrin Mac-1 regulates thrombosis via interaction with platelet GPIbα

**DOI:** 10.1038/ncomms16124

**Published:** 2017-07-04

**Authors:** Yunmei Wang, Huiyun Gao, Can Shi, Paul W. Erhardt, Alexander Pavlovsky, Dmitry A. Soloviev, Kamila Bledzka, Valentin Ustinov, Liang Zhu, Jun Qin, Adam D. Munday, Jose Lopez, Edward Plow, Daniel I. Simon

Nature Communications 8: Article number:15559 ; DOI: 10.1038/ncomms15559 (2017); Published 05 30 2017; Updated 07 04 2017

The original version of this Article contained an error in the email address of the corresponding author Daniel I. Simon. The correct email is Daniel.Simon@UHHospitals.org. The error has been corrected in the HTML and PDF versions of the article.

