# Supplementary material for: Leukocyte integrin Mac-1 regulates thrombosis via interaction with platelet GPIbα
Source: Nat Commun. 2017 May 30;8:15559. doi: 10.1038/ncomms15559 (PMC5477519; doi:10.1038/ncomms15559)
Supplement: Supplementary Information — Supplementary Figures and Supplementary Table. [file ncomms15559-s1.pdf]

Supplementary Figure 1

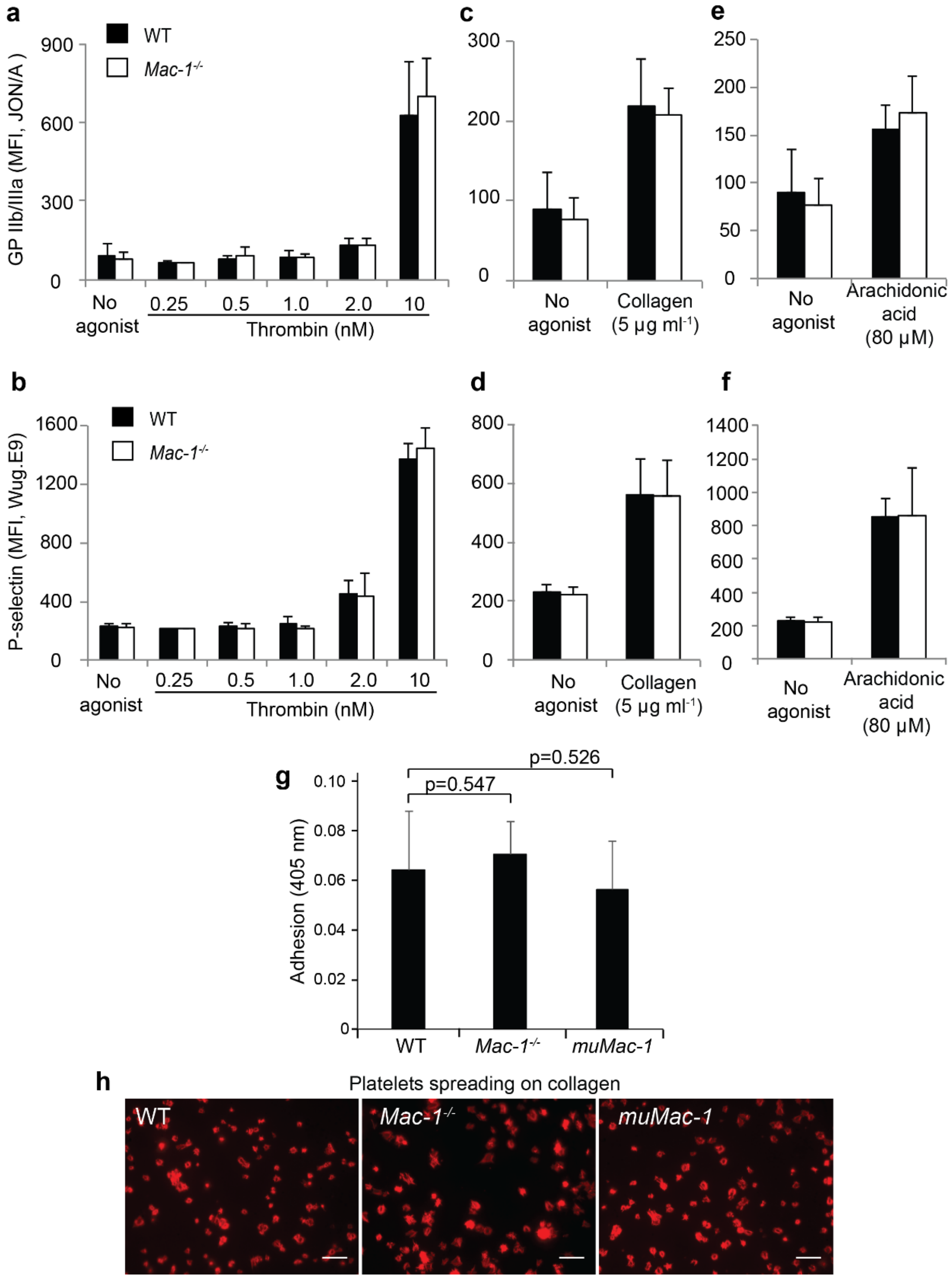

Supplementary Figure 1: Deficiency of Mac-1 has no effect on platelet activation. Platelet activation in WT and *Mac-1*$^{-/-}$ platelets. Flow cytometric analysis of platelet activation measured by the expression of GPIIb/III α (**a, c, e**) and P-selectin (**b, d, f**) using the JON/A or Wug.E9 antibody (10 µl of mixed JON/A and Wug.E9 antibodies per 25 µl platelets). Activation was assessed following stimulation of washed platelets from WT (black bars) and *Mac-1*$^{-/-}$ (white bars) mice with α-thrombin (0-10 nM), collagen (0-5 µg ml^{-1}) or arachidonic acid (0-80 µM). Platelet adhesion (**g**) and spreading (**h**) on collagen is preserved in platelets isolated from *Mac-1*$^{-/-}$ and *muMac-1* mice. Each image is representative of 8-12 images per group from two independent experiments. Data (mean fluorescence intensity, MFI or absorbance at 405 nm) were analyzed by FACS Diva 6.2 and are presented as mean ± SD, n=3. P-values are from unpaired two-tailed t-test. Scale bar: 10 µm.

Supplementary Figure 2

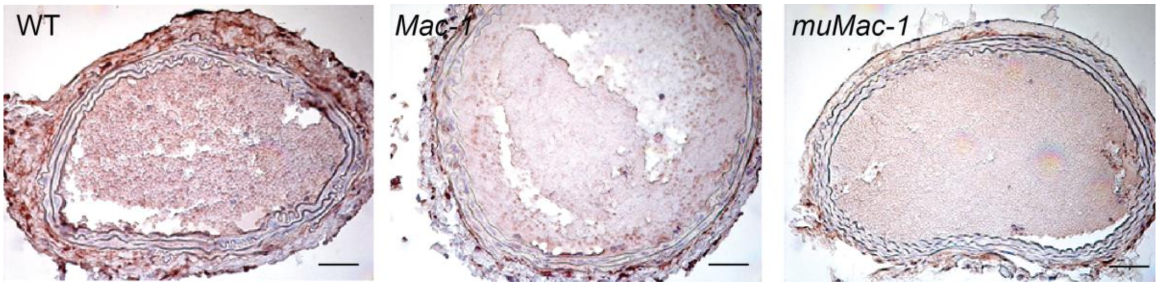

Supplementary Figure 2. Decreased expression of TF in the carotid artery and thrombi of *Mac-1*$^{-/-}$ and *muMac-1* mice after photochemical injury. Carotid arteries from WT, *Mac-1*$^{-/-}$, and *muMac-1* mice were harvested after photochemical-induced thrombosis. Standard immunohistochemical staining was carried out using anti-mouse tissue factor antibody (2 μg ml^{-1}). Each image is a representative of 4-8 images per group. Scale bar: 50 μm.

Supplementary Figure 3

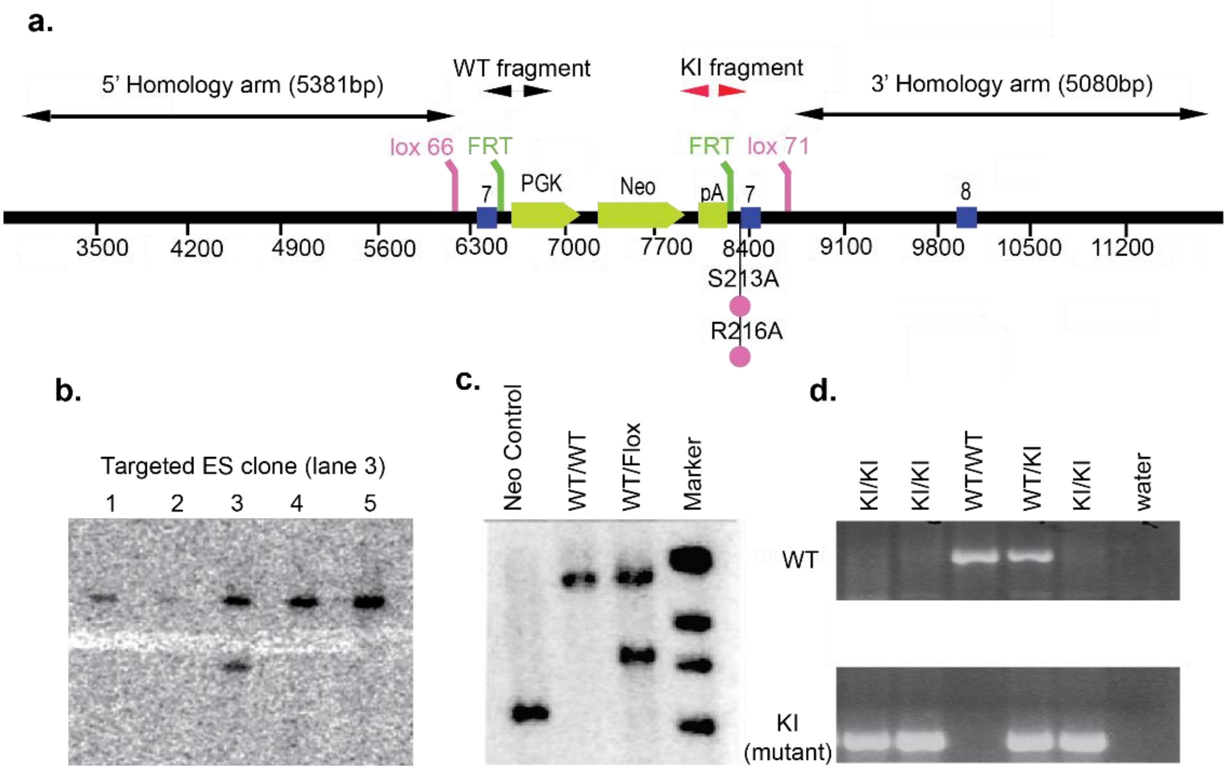

Supplementary Figure 3. **Generation of *muMac-1* ($S^{213}A/R^{216}A$) mice.** (**a**) Schematic of targeting design. Targeting vector was constructed by cloning four fragments (mutant exon 7 fragment (KI), wild-type exon 7 fragment (wt), 5’ homology arm (5H), and 3’ homology arm (H3) generated by PCR into the Ozgene plasmid PacF 10000113_A08. After confirmation of offspring of chimera x C57Bl/6 mating as wt/flox mice, three additional breeding were carried out to remove Neo, FLP and Cre cassettes and to bring the mutated exon 7 into reading frame (see method for details). (**b**) Detection of homologous recombinant in ES cells by Southern blot analysis. (**c**) Southern blot determination of genotypes. (**d**) Representative image of mouse genotyping by PCR performed with two separate reactions, one for the $S^{213}A/R^{216}A$ mutant band and another for the WT band.

Supplementary Figure 4

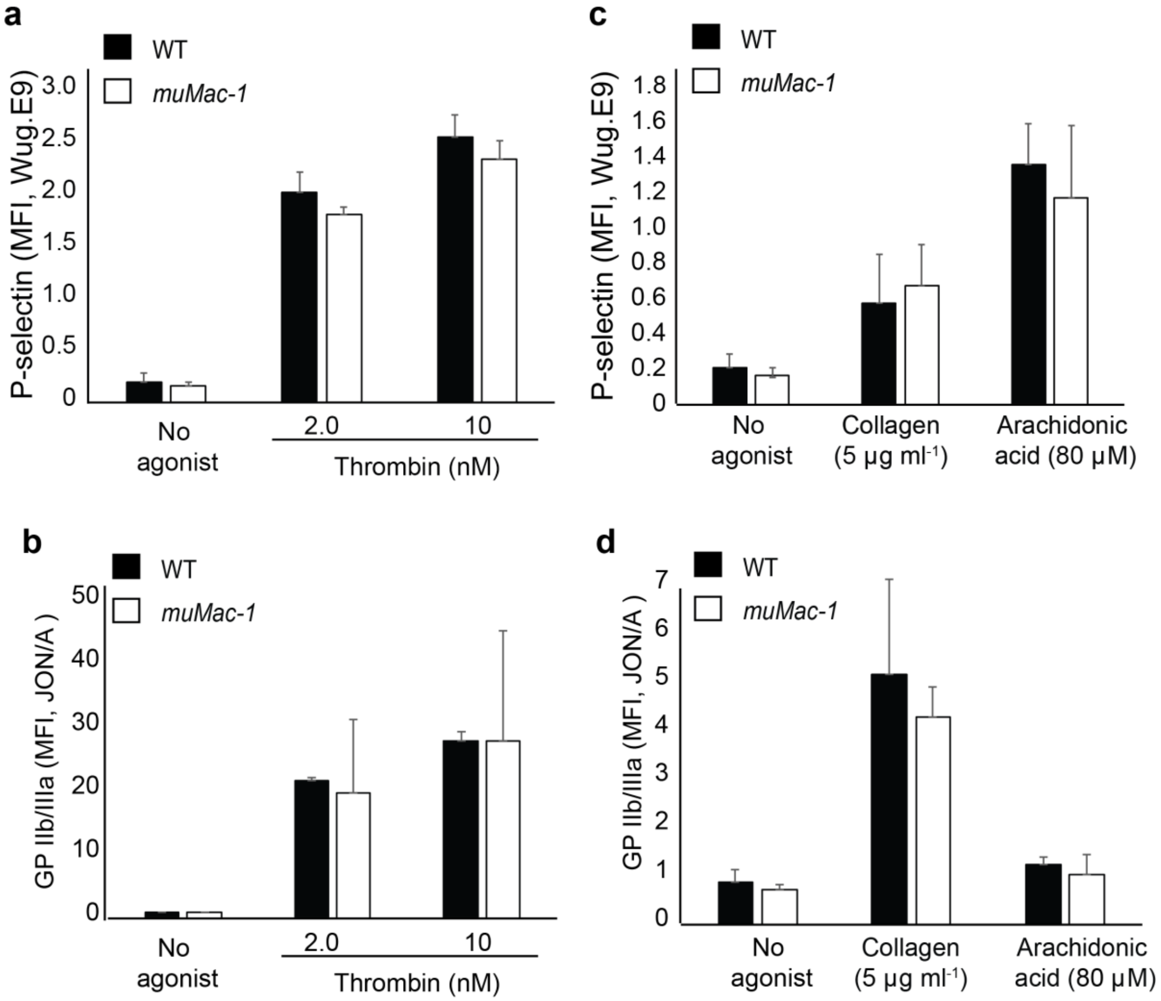

Supplementary Figure 4. ***P*latelet activation and adhesion/spreading unaffected in *muMac-1* mice.** Flow cytometric analysis of P-selectin expression (**a, c**) and GPIIb/IIIa activation assessed using staining with the Wug.E9 and the JON/A antibody (**b, d**) following stimulation of platelets from WT (black bars) and *muMac-1* (white bars) mice with 2-10 nM thrombin, 5 μg ml^{-1} collagen or 80 μM arachidonic acid. Relative mean fluorescence intensity (MFI) were analyzed with FlowJo (v.10) and presented as mean ± SD, representing one of three independent experiments.

Supplementary Figure 5

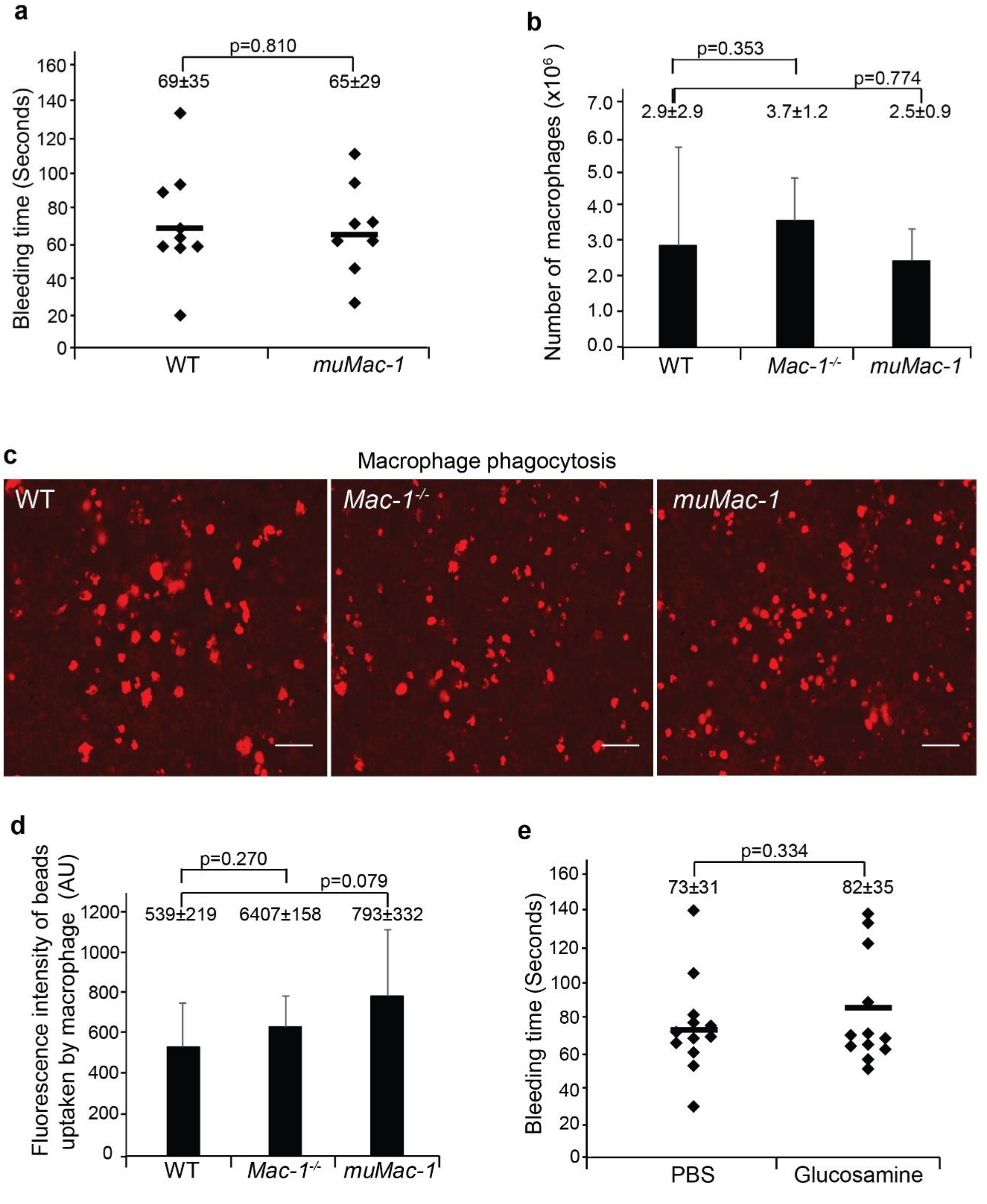

Supplementary Figure 5. Disruption of Mac-1:GPIbα complex has no influence on hemostasis and key leukocyte functions. (a) Tail bleed was assessed in WT (n=9) or *muMac-1* (n=8) mice. **(b)** Cell counts of macrophages isolated from the peritoneal cavity of mice after thioglycolate installation (n=9-10 per group). **(c)** Representative images of macrophage phagocytosis. Images are from 10-15 images per group. Scale bar: 50 μm. (**d**) Phagocytosis of fluorescent beads by macrophages from WT, *Mac-1* $^{-/-}$ and *muMac-1* mice was quantified (n=9-10 per group). AU: arbitrary units. **(e)** Tail bleed was assessed in WT mice administered intravenous PBS buffer control (n=12) or glucosamine (n=12) (27 μg per mouse). Each data point is from a single mouse. P-values are obtained from unpaired two-tailed t-test.

Supplementary Figure 6

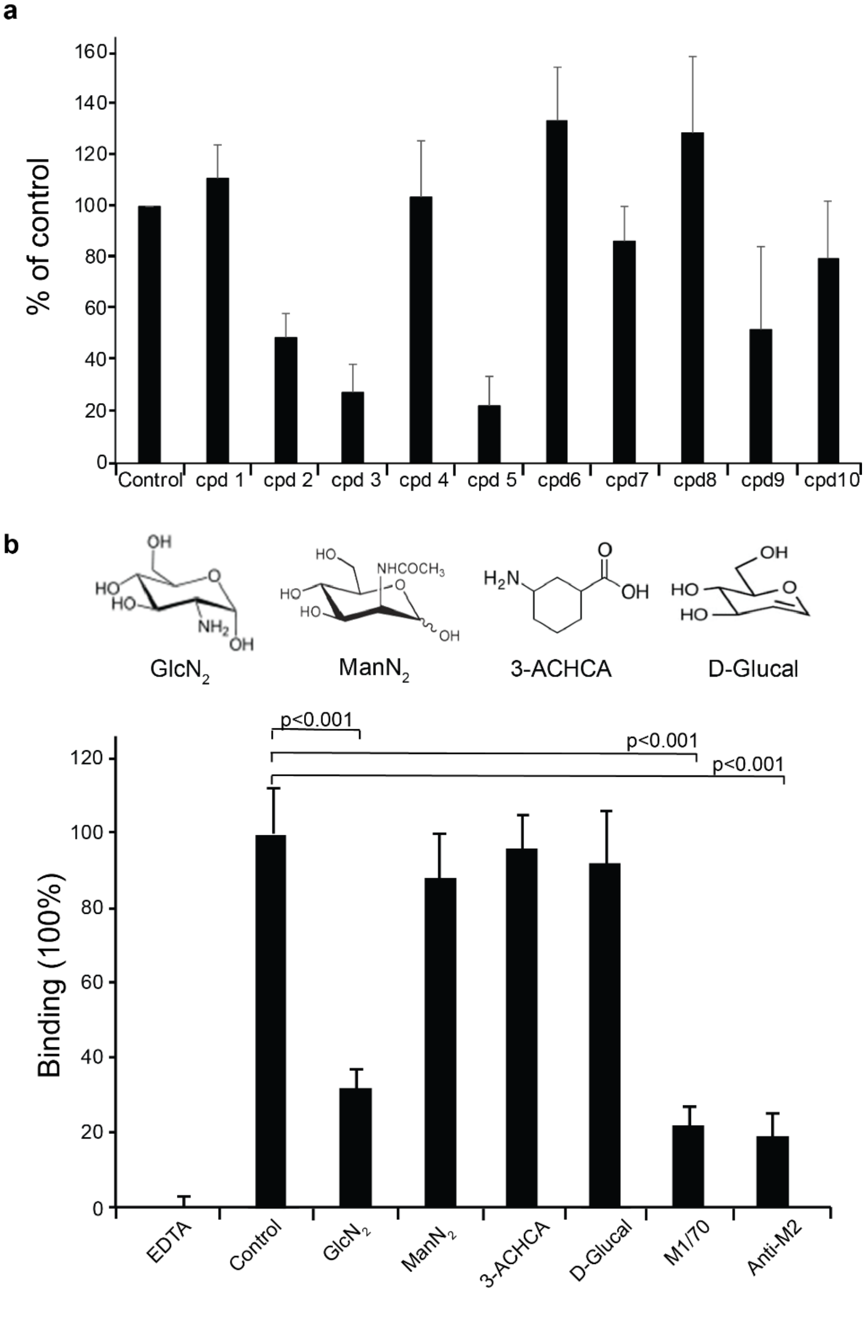

Supplementary Figure 6. Screening of small molecules that block Mac-1:GPIbα binding. (a) Representative screening assay showing the differential activity of 10 consecutive compounds on the binding of recombinant fluorescently labeled α_MI-domain to CHO cells expressing GPIbαβ. The control has no inhibitor and all compounds from the Sprectrum library were screened at a concentration of 100 µM. Background was defined as the residual binding in the presence of mAb ICRF44, and this value was subtracted from the triplicate values given by each test compound from all wells to yield the data shown. **(b)** Inhibitory activities of glucosamine ($GlcN_2$), structurally related compounds ($ManN_2$, 3-ACHCA; D-Glucal), and antibodies to α_MI-domain on the binding of α_MI-domain to GPIbα. Data (mean ± SD) are from 2-3 independent experiments, each in triplicate. P-values are from two-tailed unpaired t-test.

Supplementary Figure 7

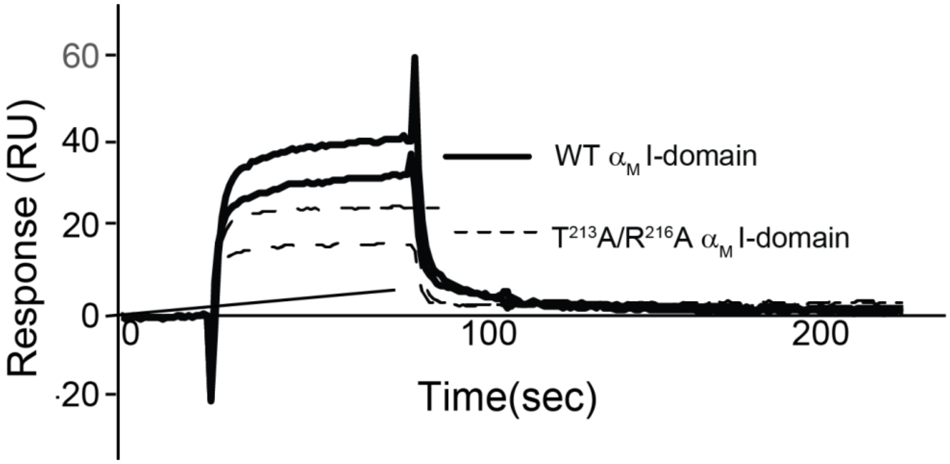

Supplementary Figure 7. Binding of glucosamine to human α_MI-domain.
The binding of glucosamine to human WT and mutant ($T^{213}A/R^{216}A$) α_MI-domain was assessed in real time by surface plasmon resonance (SPR). The coating density of the WT and α_MI-double mutant on the chip was increased from ~2500 relative units (RU) in Figure 8a on the chip to ~6000 RU on the CM5 chip surface.

Supplementary Figure 8

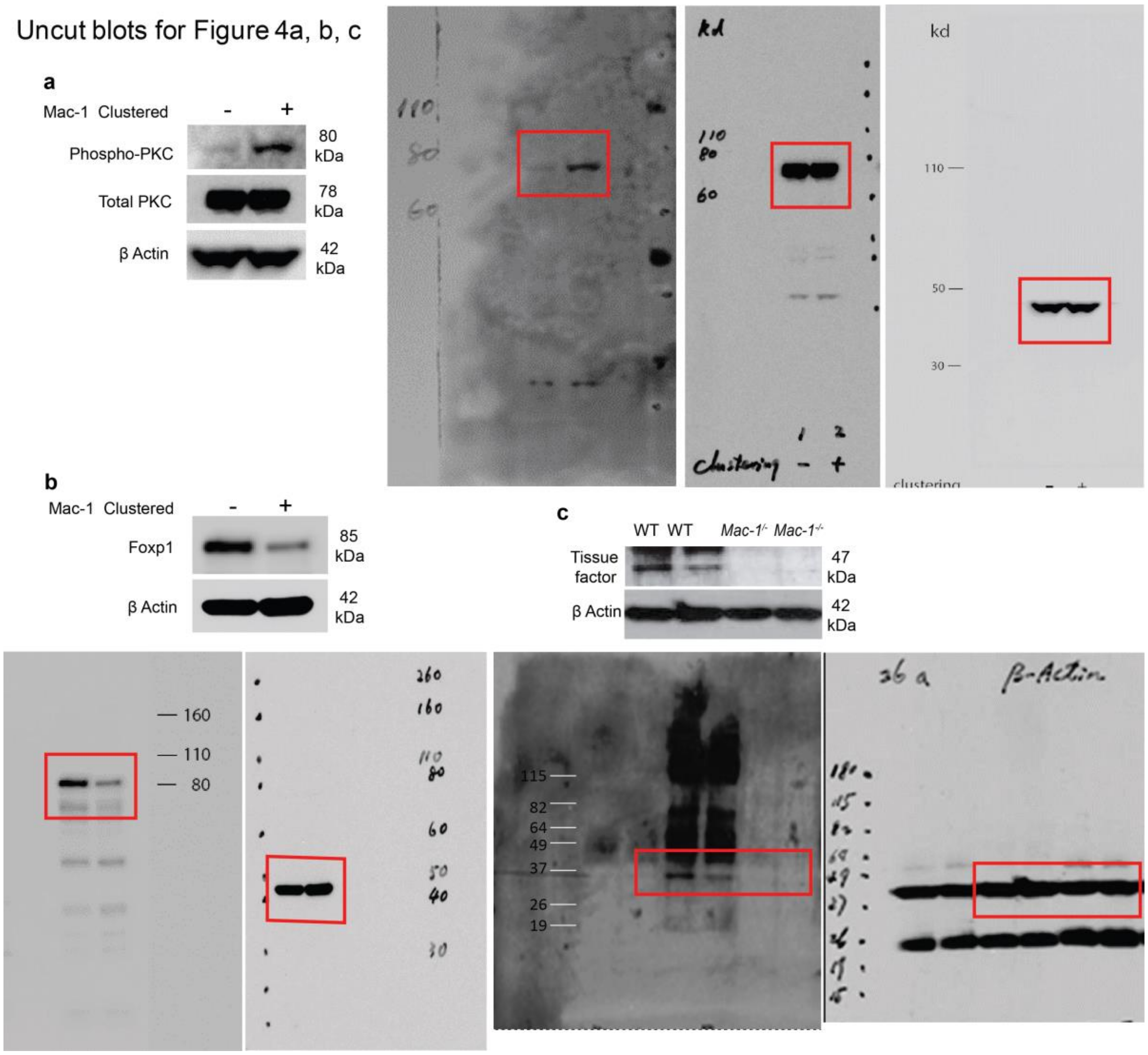

Supplementary Figure 8. Full length uncropped Western Blots for Figure 4a, b, c. (a) Phosphorylation of PKC-delta in Mac-1-clustered (+) and non-clustered (-) THP-1 monocytic cells. (b) Expression of Foxp1 in Mac-1-clustered (+) and non-clustered (-) THP-1 monocytic cells. (c) Tissue factor (TF) expression.

Supplementary Table 1 Cell counts of mouse whole blood

	WT (n=6)	*Mac1* $^{-/-}$ (n=6)		*muMac-1* (n=6)		
	Mean ± SD (k per µl)	Mean ± SD (k per µl)	P-value vs WT	Mean ± SD (k per µl)	P-value vs WT	P-value vs *Mac1* $^{-/-}$
White Blood Cells	4.4±1.6	5.4±1.7	0.320	4.7±1.3	0.719	0.448
Granulocytes	0.6±0.4	0.8±0.5	0.482	0.7±0.2	0.856	0.507
Monocytes	0.4±0.1	0.4±0.1	0.814	0.3±0.1	0.456	0.219
lymphocytes	3.4±1.1	4.2±1.1	0.256	3.8±1.1	0.609	0.491
